# H_2_/CO_2_ separations in multicomponent metal-adeninate MOFs with multiple chemically distinct pore environments†

**DOI:** 10.1039/d0sc04979d

**Published:** 2020-10-15

**Authors:** Zachary M. Schulte, Yeon Hye Kwon, Yi Han, Chong Liu, Lin Li, Yahui Yang, Austin Gamble Jarvi, Sunil Saxena, Götz Veser, J. Karl Johnson, Nathaniel L. Rosi

**Affiliations:** Department of Chemistry, University of Pittsburgh Pittsburgh PA 15260 USA nrosi@pitt.edu; Department of Chemical and Petroleum Engineering, University of Pittsburgh Pittsburgh PA 15260 USA; U.S. Department of Energy, National Energy Technology Laboratory Pittsburgh PA 15236 USA; Oak Ridge Institute for Science and Education Pittsburgh PA 15236 USA

## Abstract

Metal–organic frameworks constructed from multiple (≥3) components often exhibit dramatically increased structural complexity compared to their 2 component (1 metal, 1 linker) counterparts, such as multiple chemically unique pore environments and a plurality of diverse molecular diffusion pathways. This inherent complexity can be advantageous for gas separation applications. Here, we report two isoreticular multicomponent MOFs, bMOF-200 (4 components; Cu, Zn, adeninate, pyrazolate) and bMOF-201 (3 components; Zn, adeninate, pyrazolate). We describe their structures, which contain 3 unique interconnected pore environments, and we use Kohn–Sham density functional theory (DFT) along with the climbing image nudged elastic band (CI-NEB) method to predict potential H_2_/CO_2_ separation ability of bMOF-200. We examine the H_2_/CO_2_ separation performance using both column breakthrough and membrane permeation studies. bMOF-200 membranes exhibit a H_2_/CO_2_ separation factor of 7.9. The pore space of bMOF-201 is significantly different than bMOF-200, and one molecular diffusion pathway is occluded by coordinating charge-balancing formate and acetate anions. A consequence of this structural difference is reduced permeability to both H_2_ and CO_2_ and a significantly improved H_2_/CO_2_ separation factor of 22.2 compared to bMOF-200, which makes bMOF-201 membranes competitive with some of the best performing MOF membranes in terms of H_2_/CO_2_ separations.

## Abstract 2

Tailorable multicomponent MOFs and MOF membranes for efficient H_2_/CO_2_ separation.

## Graphical abstract

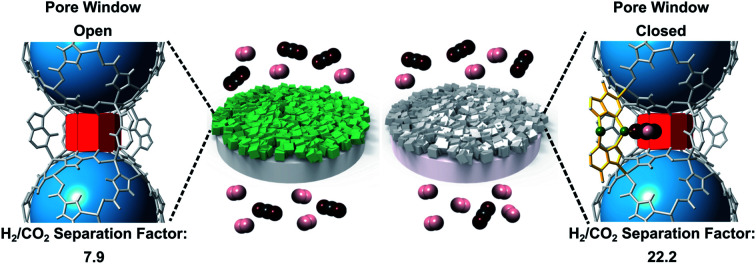

## Introduction

The vast majority of metal–organic frameworks (MOFs) consist of two components: one metal ion/cluster and one organic linker.^1–3^ In general, such MOFs have uniform pores and channels, and from the point of view of a guest molecule, all the internal space and surfaces, apart from defects, would appear identical. For many target applications,^4^ such as gas storage^5^ or catalysis,^6^ uniform pore space may be ideal. However, MOFs having more complex labyrinths of pores and channels and multiple interior regions with distinct structure and chemical functionality may lead to increasingly complex functions.^7,8^ For example, different cavities may each contain unique yet complementary reactive centres for multiple reaction steps, or different molecules may diffuse through different pathways, which could lead to improved molecular separations.

Achieving increased structural and functional complexity often involves incorporation of multiple components (*i.e.*, metals + linkers ≥ 3) into the MOF material.^9,10^ These components may be partitioned into specific domains (*e.g.*, core–shell^11–15^ and stratified MOFs^16^ or other MOF-on-MOF architectures^17–19^), or they may be distributed either randomly (*e.g.*, multivariate MOFs^20,21^) or periodically^7,8,22–38^ throughout a MOF lattice. Strategies for constructing MOFs with multiple periodically arranged components generally involve syntheses that (i) incorporate multiple linkers having different Lewis basic donor groups (*e.g.*, metal-carboxylate 2-D grids pillared by N-donor linkers^22,26,28^ or metal-adeninate-carboxylate bio-MOFs^24,29^); (ii) incorporate multiple linkers of different length and/or geometry having identical donor groups (*e.g.*, MOFs with multiple different carboxylate linkers);^7,23,25,27,34,35^ (iii) incorporate hetero-multitopic linkers such as (iso)nicotinate that preferentially coordinate different metal ions at either coordination sites;^30–33,36^ or (iv) use some combination of approaches i–iii (*e.g.*, mixing both pyrazolate and multicarboxylate linkers).^8,38^ Regardless of the approach, predicting the structural outcome of these syntheses is quite challenging and few examples of explicit structural design exist.^33,35,36^ Expanding the library of multicomponent MOFs is critical as it allows for identification of structural motifs and patterns that can then be used as input for reticular design.

Our foray into the design and construction of multicomponent MOFs began with exploration of three component metal-adeninate-carboxylate frameworks.^24,39^ These MOFs exhibit both zinc-adeninate and zinc-carboxylate motifs, providing some evidence that multiple linkers with different Lewis-basic donors could result in distinct structural motifs. Motivated by these findings and those of others,^8,30,31^ we reasoned that combining two different Lewis basic donor sites onto one ligand may provide a facile means of installing multiple different structural and functional motifs into a MOF, provided that we consider the metal coordination preferences of the different donors. Accordingly, we used isonicotinate as a linker to design and construct MOFs with up to four distinct structural components (three different metal nodes and one linker).^36^ Here, we build upon these strategies for accessing multicomponent MOFs with multiple different structural motifs and explore the structural space that results from combining adenine with the heterobifunctional linker pyrazolate. Our efforts thus far afforded two isoreticular multicomponent MOFs that exhibit multiple structurally and chemically distinct cavities and numerous possible molecular diffusion pathways. During our development of this work, one of these MOFs was reported by Li and coworkers, who studied its application in ethane/ethylene separations.^40^ Here, we examine their structures and demonstrate, both computationally and experimentally, how their unusual pore space confers a benefit in terms of H_2_/CO_2_ separations compared to various canonical two-component MOF materials. Significantly, we fabricate membranes using these MOFs, we perform membrane permeation studies, and we show that a slight structural difference between the two isoreticular analogues leads to a significant improvement in the H_2_/CO_2_ separation factor.

## Results and discussion

**Fig. 1:**
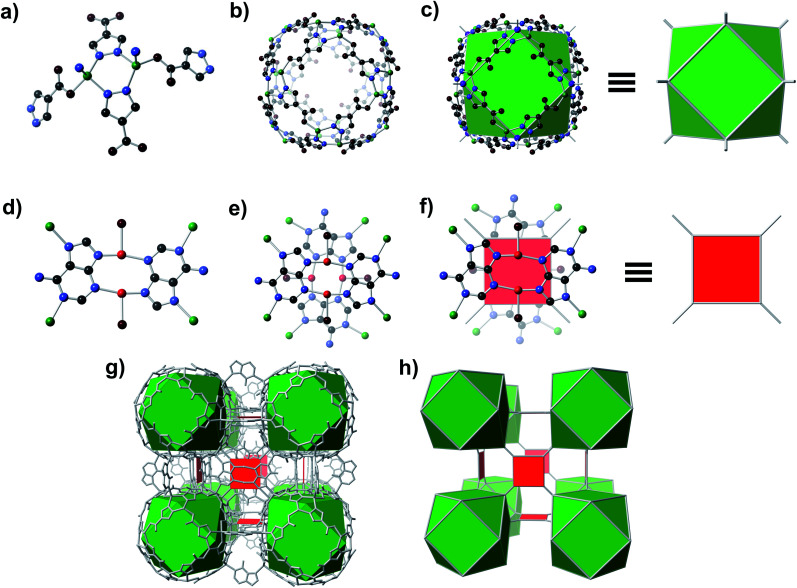
Structure of bMOF-200. (a) **Zn-pyz** dimers interconnect to create (b) pseudo-spherical cages. Connecting points located at the centre of each dimer results in (c) cuboctahedra. (d) The second structural motif consists of **Cu-ad** dimers and coordinated solvent (coordinated solvent is reduced to a single oxygen for clarity). Two of these dimers stack perpendicularly to form (e) a Cu_4_(ad)_4_(DMF)_4_ motif, which can be represented as (f) a square. (g) The bMOF-200 framework can thus be simplified to (h) the ftw-a net. Green, red, black, blue, and maroon spheres signify Zn, Cu, C, N, and O atoms, respectively. Purple spheres indicate the points of extension of the **Zn-pyz** cuboctahedra.

A solvothermal reaction of adenine, 4-pyrazolecarboxylic acid, and divalent Zn and Cu salts in dimethylformamide (DMF) afforded slightly yellow cubic crystals, which, according to single crystal X-ray diffraction (SC-XRD) studies (see ESI,† Section S7), adopt the cubic space group *Fm*3̄*c* with unit cell dimensions *a* = *b* = *c* = 43.62 Å.† The structure consists of Zn-pyrazolate (**Zn-pyz**) cages interlinked by Cu-adeninate (**Cu-ad**) motifs. The **Zn-pyz** cages are constructed from **Zn-pyz** dimers (Fig. 1a) in which two tetrahedral Zn(ii) are bridged by the N–N linkages of two **pyz**; the remaining two sites on each Zn(ii) are occupied by a monodentate carboxylate from a **pyz** on a neighbouring **Zn-pyz** motif and either N1 or N7 from an **ad** in a neighbouring **Cu-ad** dimer. Twelve **Zn-pyz** dimers link together to form the **Zn-pyz** cages (Fig. 1b). We note that each individual **Zn-pyz** cage is chiral and alternating cages have opposite chirality, resulting in an achiral crystal. The underlying net of the **Zn-pyz** cages is perhaps best simplified by treating each **Zn-pyz** dimer as a single point (purple sphere in Fig. 1c); connecting these points yields a cuboctahedron (Fig. 1c). The **Cu-ad** motifs consist of two planar Cu_2_ad_2_(DMF)_2_ dimers stacked perpendicular to one another with an interplanar distance of ∼3.4 Å (Fig. 1d and e). Each planar dimer is constructed from two Cu(i) bridged by two **ad** through N3 and N9; the Cu(i) are terminally coordinated to one DMF. The intercopper distance in each dimer is 2.86 Å, which is within the range of Cu(i)–Cu(i) bonding.^24^ Each stacked dimer has eight points of extension, two from each **ad** at N1 and N7. The stacked dimers link four **Zn-pyz** cages together, with two linkages to each cage. If we simplify each stacked dimer as a 4-connected planar structural motif and connect the corners to the centroid (purple sphere) of the **Zn-pyz** dimers, the net reduces to ftw-a (Fig. 1f–h).^25^ The actual net, of course, is more complex, but visualization as ftw-a, we believe, is instructive. We name this material bio-MOF-200 (bMOF-200) and install it as another member of the metal-adeninate bio-MOF series.^24,39,41,42^

The molecular formula of bMOF-200, Zn_2_Cu_1_(pyz)_2_(ad) (DMF)·2DMF, H_2_O, was determined through examination and comparison of SC-XRD, elemental analysis (EA), ^1^H nuclear magnetic resonance (^1^H NMR) spectroscopy, and thermogravimetric analysis (TGA) data, collectively (see ESI for details; Fig. S1, S2 and Section S7†). It is known that Cu(ii) can be reduced *in situ* during solvothermal reactions in DMF.^36^ Upon exposure to air, the crystals transition from colourless to light green, presumably due to Cu(i) → Cu(ii) oxidation. Assignment of Cu(i) was verified in part based on X-ray photoelectron spectroscopy, which shows evidence of Cu(ii) on the surface, but upon etching, Cu(i) in the bulk (Fig. S3†). Continuous wave electron pair resonance (CW-EPR) confirmed the presence of Cu(ii) upon exposure of the material to atmosphere (Fig. S4†). The CW-EPR spectrum was simulated with two components, indicating two distinct Cu(ii) coordination environments. One component was a broad, featureless signal, likely resulting from dipolar or exchange broadening which occurs when two or more Cu(ii) ions are in close proximity.^43^ The second component had well resolved features from hyperfine interactions. The spectral parameters are available in the ESI.† These EPR parameters are consistent with four-coordinate square planar.^44^ Alternatively, the spectrum is also generally consistent with recorded three-coordinate Cu(ii) species in T- and Y-shaped geometries.^45–47^ These EPR findings support the expected geometry of the Cu(ii) centre. The powder X-ray diffraction (PXRD) pattern (Fig. S5†) of as-synthesized bMOF-200 matches the pattern simulated from SC-XRD, confirming the phase purity of the product material.

**Fig. 2:**
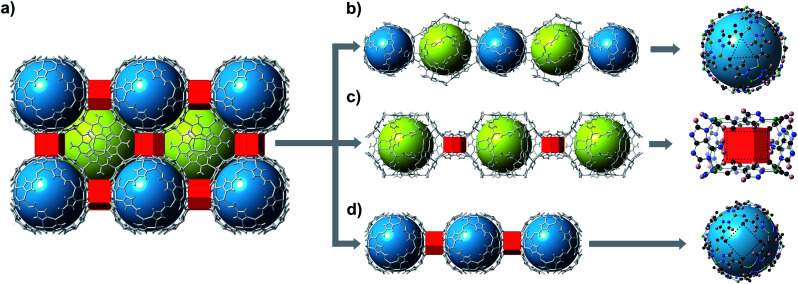
Potential diffusion pathways through bMOF-200. (a) bMOF-200 structure where blue spheres, yellow spheres, and red prisms correspond to P1, P2, and P3, respectively. Pore windows are indicated by dotted lines (right). (b) Pathway P1 → P2 is limited by a triangular pore window (∼2.1 Å). (c) A rectangular window measuring (∼4.0 × 2.6 Å) connects P2 and P3. (d) The largest aperture of ∼4.3 Å is the square window between P1 and P3.

bMOF-200 exhibits three distinct cavities, hereafter referred to as P1, P2, and P3 (Fig. 2a). P1 is within the **Zn-pyz** cage. P2 is the cubic cavity defined by eight **Zn-pyz** cages at the corners of a cube and six Cu_2_ad_2_(DMF)_2_ dimers on the faces. Both P1 and P2 measure ∼15.5 Å in diameter. A triangular window (∼2.1 Å) sitting on the triangular face of the **Zn-pyz** cage links P1 to P2. The square windows (∼4.3 Å) on the square faces of the **Zn-pyz** cages open into the third pore space, P3, which sits between two adjacent **Zn-pyz** cages. P3 is a small prismatic cavity measuring ∼7 Å in diameter and is decorated with four amino groups from the adeninates. P3 shares a rectangular pore window (∼4.0 × 2.6 Å) with P2, which, in the as-synthesized material, is obstructed by the coordinated DMF molecule. The connectivity of the pores and the resulting diffusive pathways of guest molecules along with pore windows are shown in Fig. 2b–d. The P1 → P2 pathway requires passage through the triangular pore window, and would only be accessible to gases with exceptionally small kinetic diameters (Fig. 2b). In the second pathway (Fig. 2c), molecules would pass through the pore window connecting P2 and P3. The most accessible pathway is along P1 and P3, where molecules would pass through the comparatively larger square pore window connecting P1 and P3 (Fig. 2d). We recognize that the diffusive pathways shown in Fig. 2 represent only the simplest paths through a maximum of two types of pores. In reality, more complex diffusion paths are likely; however, these are much more difficult to conceptualize and model. Thus, we determined that limiting our investigation to the simplest pathways would provide sufficient molecular diffusion information through bMOF-200.

We conducted gas adsorption studies to determine the porosity of bMOF-200 and, more specifically, the accessible pore space given the limiting pore window diameters. Gas adsorption studies were performed on methanol-exchanged material, MeOH-bMOF-200† (see ESI† for experimental details and characterization including SC-XRD, PXRD, EA, NMR, Fourier transform infrared spectroscopy, and TGA, Fig. S6–S9† as well as sample activation procedures). We first collected N_2_ (kinetic diameter = 3.6 Å) isotherms at 77 K (Fig. S13†) from which we calculated a Brunauer–Emmett–Teller (BET) surface area of 1317 m^2^ g^−1^, which accounts for approximately 50% of the theoretical value of ∼2400 m^2^ g^−1^ determined using Connolly surface area modelling. This possibly indicates that N_2_ can only access P1 and P3 with only a small amount entering P2. Comparatively, the CO_2_ (kinetic diameter = 3.3 Å) isotherm collected at 195 K yielded a BET surface area of 1741.37 m^2^ g^−1^, correlating to 74% of the corresponding theoretical surface area (Fig. S14†), indicating that CO_2_ can access at least a portion of P2. These results, combined with the complex pore circuitry, suggest that molecular sieving is possible in bMOF-200.

**Fig. 3:**
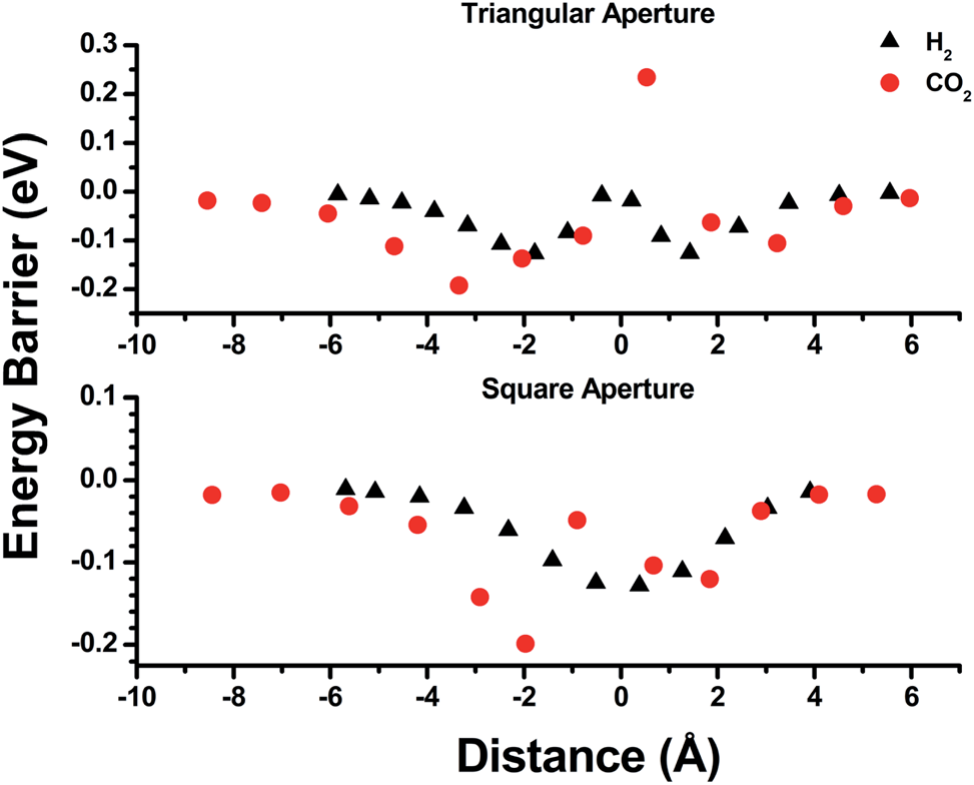
Energy barrier calculations for the triangular (top) and square (bottom) pore windows of P1. The distance values of −9, 0, and 6 Å correspond to the centre of P1, the respective pore window, and the exterior of P1 (toward vacuum).

Empirical determination of candidate binary gas separations for a given MOF is time consuming and laborious. Due to their crystalline nature, MOFs are highly amenable to computational investigation. To identify a binary gas mixture that might be ideally separated into its individual components using bMOF-200, we used Kohn–Sham density functional theory (DFT) in conjunction with the climbing image nudged elastic band (CI-NEB) method^48,49^ to study the diffusion path of various gases through the square and triangular apertures of the isolated **Zn-pyz** cage (P1) (Fig. 3; see ESI† for details). We assigned the starting point as −9 Å, which corresponds to the centre of P1. Generally, there is an attractive interaction between the guest molecule and the atoms near the apertures (0 Å). This interaction is similar both in strength and distance to the calculated physisorption inside the MOF. In the case of H_2_, the interaction is the strongest near the centre of the square aperture between P1 and P3, and there is only a slight barrier of 0.1 eV to travel across the smaller triangular aperture (P1 to P2). In contrast, the barrier is much higher (0.43 eV) for CO_2_ to diffuse through the triangular opening into P2. Additionally, CO_2_ also has to overcome a barrier of 0.15 eV to move across the square opening. Overall, our calculations predict that CO_2_ diffuses more slowly than H_2_. More importantly, our calculations show that the diffusion path P1 → P2 through the triangular apertures is significantly more energetically accessible for H_2_ and hindered for CO_2_. Therefore, if CO_2_ were to enter into P2 it would have to occur through P3.

Further DFT studies were conducted to calculate binding energies of H_2_ and CO_2_ in bMOF-200 at various sites within a static framework model (Table S1†). The strength of interaction of both guest molecules with bMOF-200 falls within the range of physisorption, and no chemisorption or strong binding sites were found. Overall, bMOF-200 interacts more strongly with CO_2_ than H_2_ by approximately 0.1 eV. This value suggests that the first layer of physisorption is likely dominated by CO_2_ by roughly 50 : 1 at room temperature. Furthermore, the distance for physisorption fell within a narrow window of 2.4 Å to 3.2 Å, which suggests that guest molecules likely travel in single file fashion through all apertures. Diffusion in this manner is likely to occur inside P3, where CO_2_ interacts most strongly (0.3 eV) with the MOF (Fig. S15†). Four primary amines from four adeninates line P3 creating a Lewis basic environment that acts as a trap for CO_2_ as they pass single file through the aperture between P1 and P3. In contrast, H_2_ does not appear to experience strong local interactions at P3 and prefers to bind closer to the Cu sites. Thus, the diffusion through P3 to P2 is likely faster for H_2_ than CO_2_. We predicted that the combination of slow diffusion of CO_2_ through the MOF due to small pore windows and the strong adsorptive interactions with the amine functionalized P3 would make bMOF-200 an effective material for H_2_/CO_2_ separations. Pure H_2_ is used heavily in chemical industries and is produced predominately by steam reforming of natural gas.^50^ The main by-product of this process is CO_2_, which can be present in as high as 15% by mass before purification.^51^ Purification of gases in the production of chemical feedstock is typically done by cryogenic distillation, an expensive and energy intensive process.^52^ Sorbents, MOFs and ZIFs in particular, have been explored as attractive low-cost alternatives to distillation for H_2_/CO_2_ separations.^53–64^

**Fig. 4:**
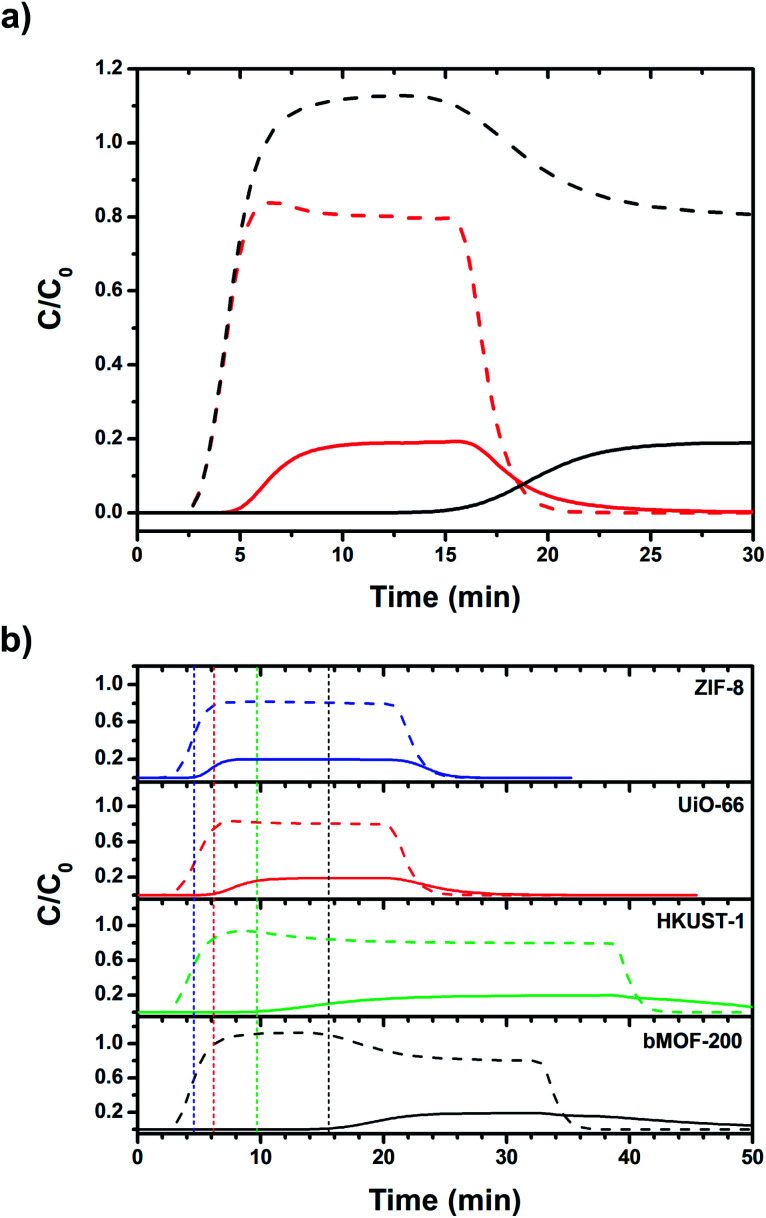
(a) Variable column length breakthrough curves of bMOF-200 (red: 10 mm; black: 50 mm). (b) Breakthrough curves of bMOF-200 (black), HKUST-1 (green), UiO-66 (red), and ZIF-8 (blue) at constant column length of 50 mm. In both plots, dashed lines correspond to H_2_ and solid lines correspond to CO_2_. Vertical dotted lines correspond to the time at *C*/*C*_0_ = 0.01 for CO_2_. Abrupt drops in *C*/*C*_0_ correspond to switching to pure carrier gas.

To test the H_2_/CO_2_ separation capabilities of bMOF-200, we first conducted column breakthrough experiments. A feed gas ratio of 80 : 20 H_2_ : CO_2_ was selected as an approximate to the relative ratios after industrial scale water gas shift (WGS) reactions using methane as the feedstock.^65^ Other gases such as N_2_, CH_4_, and CO, are only present in small amounts in the product stream of the WGS reaction. Two columns were packed with dried bMOF-200 to column lengths of 10 and 50 mm to study the effect of column length. The feed gas was introduced to the packed columns at room temperature and the resulting breakthrough curves were collected (Fig. 4a). The H_2_ breakthrough time was consistent for both column lengths, indicating very weak interactions between bMOF-200 and H_2_. However, the CO_2_ breakthrough time is positively dependent on column length, where more bMOF-200 material leads to longer breakthrough times. Furthermore, a “roll-up”, or overshoot, behaviour is observed in the H_2_ curves for longer column lengths, which corresponds to the displacement of adsorbed H_2_ by CO_2_,^66,67^ in accordance with our computational studies. These results illustrate the promise of bMOF-200 as a solid sorbent for H_2_/CO_2_ separation. Another series of breakthrough experiments were performed to compare bMOF-200 to canonical MOFs bearing similar features to bMOF-200: ZIF-8 ^68^ because of its small pore windows; UiO-66 ^69^ because of its multiple pore diameters; and HKUST-1 ^70^ because of its unsaturated Cu(ii) sites (see ESI for synthesis and characterization details; Fig. S16 and Table S2†). The breakthrough results for all four MOF materials of equivalent column lengths are shown in Fig. 4b. The vertical dotted lines correspond to the timepoint where the relative concentration, *C*/*C*_0_, of CO_2_ in the effluent is equivalent to 0.01, which we designate as the breakthrough point. All four MOFs exhibit breakthrough times of approximately 2.5 min for H_2_ due to weak adsorbate–adsorbent interactions and the small kinetic diameter of H_2_ (2.9 Å) compared to the respective pore window diameters for each MOF. Of the four MOFs, bMOF-200 demonstrated the best H_2_/CO_2_ separation capability with a CO_2_ breakthrough time of 15.5 min. The “roll-up” feature is again observed in the H_2_ curve for bMOF-200 as well as HKUST-1. In each breakthrough experiment (Fig. 4), abrupt drops in *C*/*C*_0_ correspond to switching to pure carrier gas once equilibrium was achieved. To summarize, bMOF-200 shows the strongest interactions with CO_2_ among the tested MOFs, a desirable property for H_2_ purification applications.

**Fig. 5:**
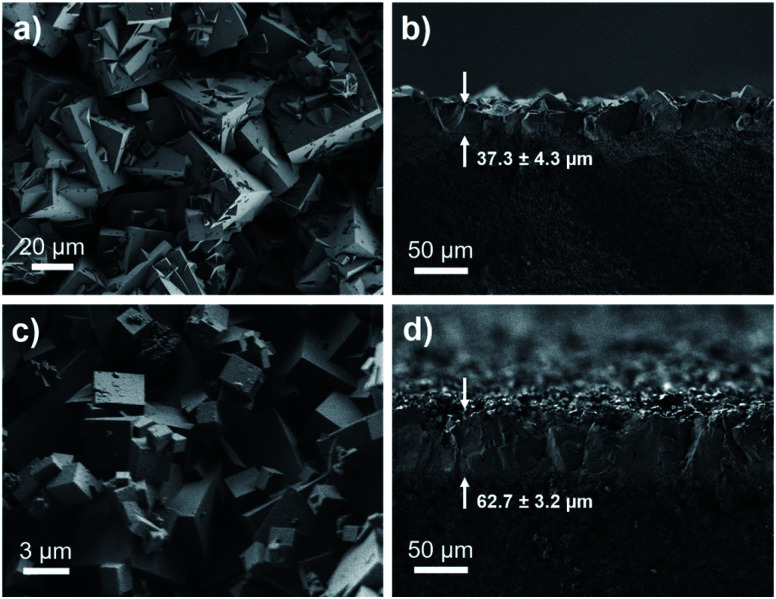
SEM images of top-view and cross-sectional view of bMOF-200 membrane (a and b) and bMOF-201 membrane (c and d). The membrane thicknesses are shown with arrows in (b and d).

Membrane technologies hold great promise for the chemical separations industry.^52^ Therefore, we fabricated membranes of bMOF-200 to further investigate its gas separation capabilities and to benchmark it against other MOF membranes for H_2_/CO_2_ separations. bMOF-200 membranes were grown solvothermally on α-alumina substrates using a seeded growth approach^71–74^ (see ESI for details; Fig. S17†). Scanning electron microscopy (SEM) images of the membranes reveal intergrown cubic crystals of bMOF-200 (Fig. 5a). The average membrane thickness (∼37.3 ± 4.3 μm) was measured from images of fractured membranes acquired at 90° relative to the surface (Fig. 5b). We constructed our own binary gas permeation system for activating and testing membrane gas separations using flow controllers and a gas chromatograph with a thermal conductivity detector (Scheme S1†). For an equimolar H_2_/CO_2_ mixture, the average H_2_ permeability was 5.51 × 10^−11^ ± 6.66 × 10^−13^ mol m (m^2^ s Pa)^−1^ (164 600 Barrer) with a H_2_/CO_2_ separation factor of 7.9 ± 1.3 (Table S3†), which exceeds the corresponding Knudsen factor (4.7) and indicates molecular sieving.

**Fig. 6:**
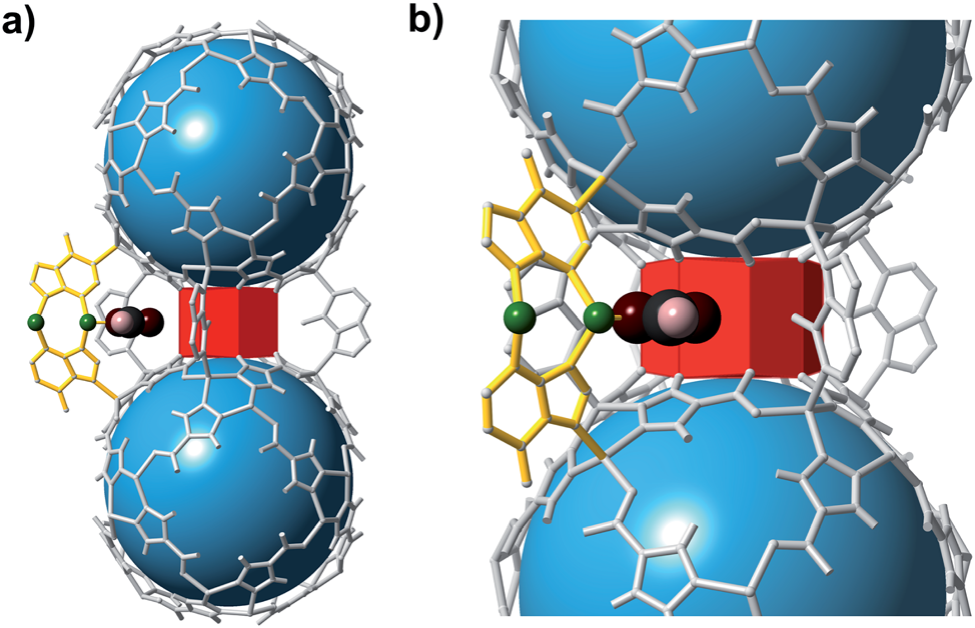
Fragments of bMOF-201 highlighting the structural differences to bMOF-200. (a) Two **Zn-pyz** cages bridged by four adeninates (grey) and one **Zn-ad** (yellow). (b) A magnified view of P3 reveals the bent nature of **Zn-ad** and the location of coordinated monocarboxylate ions (*e.g.*, formate or acetate) within the P2 → P3 pore window. Green, black, maroon, and pink atoms correspond to Zn, C, O, and H atoms, respectively.

Small modifications to the bMOF-200 structure might lead to significant changes, and possible improvement, of its H_2_/CO_2_ separation ability. Given its complex structure, adjusting the metrics of bMOF-200 *via* typical MOF methods (*e.g.* lengthening linkers, *etc.*) was not straightforward. However, we reasoned that a more subtle change, such as replacing the Cu site with Zn(ii) might be feasible. By slightly altering the synthetic conditions and removing the Cu source, we were able to synthesize the Zn only analogue, which we name bio-MOF-201 (bMOF-201). bMOF-201† is nearly identical to bMOF-200 (structure and composition data are available in the ESI; Fig. S10–S12 and Section S7†) in all aspects except the **Cu-ad** motif is now replaced by a **Zn-ad** motif. The consequences of this substitution stem from the tetrahedral geometry of the Zn(ii) centres in **Zn-ad**. Whereas the N–Cu–N angle in bMOF-200 is 167°, the comparable N–Zn–N angle in bMOF-201 is 111° with the Zn(ii) projecting into P2 (Fig. 6a). Therefore, based on analysis of the crystal structure, the size of P2 is reduced to ∼12.2 Å in the desolvated MOF. The Zn(ii) in **Zn-ad** is charge-balanced by one adeninate and a coordinated formate/acetate ion, the latter of which resides in the pore window between P2 and P3 (Fig. 6b). The resulting diameter of the pore window connecting P2 and P3 is decreased to ∼0.9 Å. We postulate that the coordinated monocarboxylate will obstruct the P2/P3 window and hinder diffusion of both H_2_ and CO_2_, which could possibly affect the H_2_/CO_2_ separation factor.

Membranes of bMOF-201 were synthesized following a similar solvothermal procedure as for bMOF-200 (see ESI for details; Fig. S18†), and SEM images reveal well-intergrown crystals and a membrane thickness of ∼62.7 ± 3.2 μm (Fig. 5c and d). The average permeability of H_2_ through bMOF-201 membranes was 3.94 ± 0.13 × 10^−11^ mol m (m^2^ s Pa)^−1^ (117 700 Barrer), which is a decrease of ∼28% compared to bMOF-200. However, the CO_2_ permeability decreased by ∼68% in bMOF-201, resulting in a 3-fold increase in separation factor to 22.2 ± 2.2 (Table S3†). We attribute these lower permeability values and the improved separation factor to the occlusion of the P3 → P2 pore window. Further, in bMOF-201, the volume of P3 decreases by approximately 11% relative to bMOF-200, which may lead to stronger interactions between CO_2_ and the Lewis basic amino groups from adeninate which line this cavity.

**Fig. 7:**
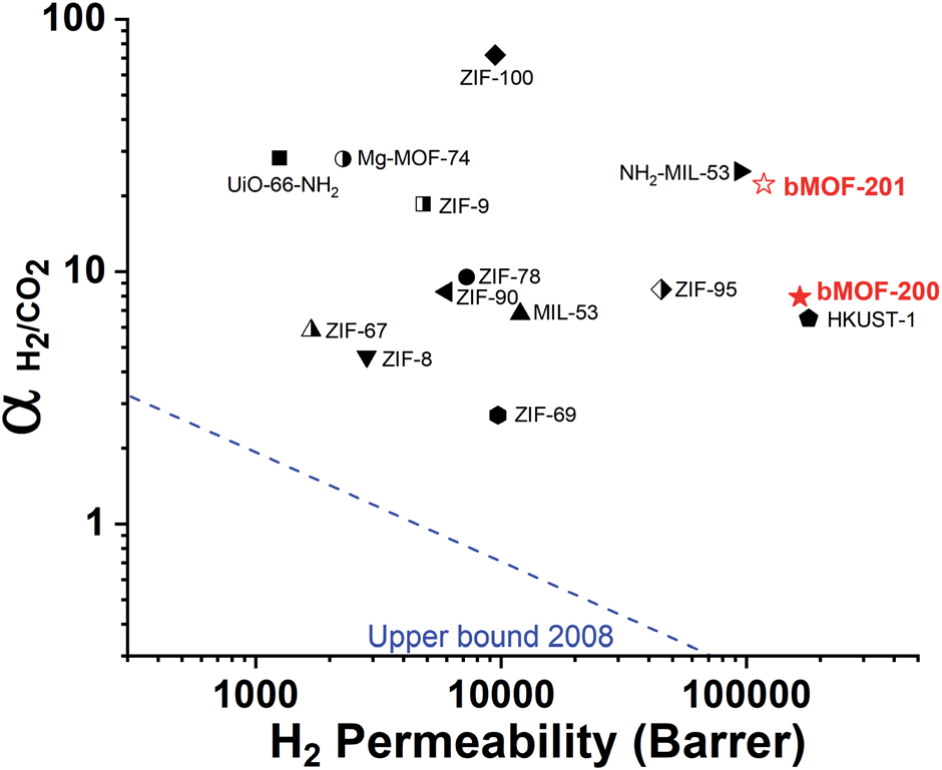
2008 Robeson plot for H_2_/CO_2_ separations using polymer membranes. Data for reported MOF membranes and bMOF-200 (filled red star) and bMOF-201 (empty red star) membranes are included for comparison. Data for other MOFs and ZIFs from ref. 53–64.

Our experimental and computational data indicate that both competitive adsorption and molecular sieving, two of the three mechanisms highlighted by Carreon,^75^ are responsible for the separation performance of bMOF-200 and bMOF-201 membranes, which exceed the 2008 Robeson upper bound for polymeric membranes (Fig. 7).^76^ The H_2_/CO_2_ separation factor of the bMOF-201 membrane is superior to other MOF membranes (HKUST-1,^53^ ZIF-69,^54^ ZIF-8,^60^ and ZIF-67 ^61^) for the same equimolar mixture and under the same testing conditions, while ZIF-100 membranes exhibit separation factors as high as 72, likely due to very efficient molecular sieving.^58^ The H_2_ permeability of bMOF-200 and bMOF-201 are amongst the highest in the comparison group, rivalling those observed for HKUST-1 ^53^ and NH_2_-MIL-53.^63^ Large H_2_ permeability values are desirable because they could ultimately reduce membrane area and make the entire gas separation module more compact. We attribute the high H_2_ permeability to diffusive pathways that are exclusively available to H_2_. Since CO_2_ strongly adsorbs within P3, it limits diffusion along P1 → P3 and along P3 → P2. H_2_, however, can also diffuse along P1 → P2, a pathway not available to CO_2_.

## Conclusion

We have prepared two isoreticular multicomponent MOFs, bMOF-200 and 201, that each contain multiple pore environments and potential molecular diffusion pathways. Through DFT calculated physisorption energy and diffusion barriers, we predicted that bMOF-200 could perform well as a solid sorbent for H_2_/CO_2_ separations based on the respective diffusivities and adsorbate–adsorbent interactions for each gas. Column breakthrough and membrane permeation studies revealed efficient H_2_/CO_2_ separation using bMOF-200, with membranes exhibiting a H_2_/CO_2_ separation factor of 7.9. Replacing a planar Cu site in bMOF-200 with a tetrahedral Zn(ii) in bMOF-201 leads to occlusion of one pore window and a decrease in the number of possible diffusion pathways for both H_2_ and CO_2_. A consequence of this structural change was significantly improved separation performance: the H_2_/CO_2_ separation factor for bMOF-201 membranes was 22.2, nearly three times greater than bMOF-200 membranes. bMOF-201 membranes outperform those of other reported MOFs when considering both H_2_ permeability and H_2_/CO_2_ separation factor.

## Conflicts of interest

There are no conflicts to declare.

## Supplementary Material

SC-011-D0SC04979D-s001

SC-011-D0SC04979D-s002
