# Supplementary material for: H2/CO2 separations in multicomponent metal-adeninate MOFs with multiple chemically distinct pore environments
Source: Chem Sci. 2020 Oct 15;11(47):12807–15. doi: 10.1039/d0sc04979d (PMC8163211; doi:10.1039/d0sc04979d)
Supplement: SC-011-D0SC04979D-s001 [file SC-011-D0SC04979D-s001.pdf]

Electronic Supplementary Material (ESI) for Chemical Science.
This journal is © The Royal Society of Chemistry 2020

Supporting Information for

H_2/CO_2 Separations in Multicomponent Metal-Adeninate MOFs with Multiple Chemically Distinct Pore Environments

Zachary M. Schulte[a], Yeon Hye Kwon[a], Yi Han[a], Chong Liu,[a] Lin Li[b], Yahui Yang[b], Austin Gamble Jarvi,[a] Sunil Saxena,[a] Götz Veser[b], J. Karl Johnson[b], and Nathaniel L. Rosi[a,b,c,d,*]

[a] Department of Chemistry, University of Pittsburgh, Pittsburgh, Pennsylvania 15260, United States

[b] Department of Chemical and Petroleum Engineering, University of Pittsburgh, Pittsburgh, Pennsylvania 15260, United States

[c] U.S. Department of Energy, National Energy Technology Laboratory, Pittsburgh, Pennsylvania 15236, United States

[d] Oak Ridge Institute for Science and Education, Pittsburgh, Pennsylvania 15236, United States

Contents:

1. General procedures…S4
   1.1 Materials…S4
   1.2 General characterization techniques…S4
2. Material preparation and characterization…S8
   2.1 Synthesis and characterization of **as-synthesized bMOF-200**…S8
   2.2 Solvent exchange procedures…S15
   2.3 Synthesis and characterization of **bMOF-201**…S20
3. Gas adsorption studies…S25
   3.1 Activation of **MeOH-bMOF-200**…S25
   3.2 Gas adsorption isotherms…S25
4. Computational methods…S28
   4.1 Computational methodology…S30
   4.2 Density functional theory (DFT) details…S31
5. Breakthrough experiments…S32
   5.1 Description of breakthrough experiments…S32
   5.2 Synthesis and characterization of reference MOFs…S33
6. Membrane fabrication and gas separation studies…S35
   6.1 Fabrication of **MeOH-bMOF-200** Membranes…S35
   6.2 Fabrication of **bMOF-201** Membranes…S35
   6.3 Membrane Characterization…S36
   6.4 Experimental Setup…S38

7. Crystallographic tables……………………………………………………………S41

7.1 Tables for **as-synthesized bMOF-200**…………………………………...S41

7.2 Tables for **MeOH-bMOF-200**………………………………...………S50

7.3 Tables for **bMOF-201**……………………………………………….…...S59

1. General procedures

1.1 Materials

$Zn(NO_3)_2 \cdot 4H_2O$, $Zn(COOCH_3)_2 \cdot 2H_2O$, $Cu(NO_3)_2 \cdot 2.5H_2O$, $Cu(Cl)_2$ (anhydrous), dimethylformamide (DMF), 70% HBF_4, and acetic acid were purchased from Sigma-Aldrich. Adenine and $ZnCl_2$ (anhydrous) were purchased from Alfa-Aesar. Methanol was purchased from Fisher Scientific. 4-pyrazolecarboxylic acid (H_2-pyz) was purchased from Accela. NANOpure® (Thermo Scientific, > 18.2 MΩ · cm) water was used in the synthesis of all MOFs. All purchased chemicals were used without further purification except where otherwise noted.

1.2 General characterization techniques

^{1}H nuclear magnetic resonance (NMR) spectra were obtained using a Bruker Advance 500 MHz spectrometer. Chemical shifts are in parts per million using the residual solvent peak (DMSO-d6, 2.5 ppm) as reference. To prepare MOF samples for NMR, approximately 1 mg of dried MOF sample was placed in a 1.5 mL Eppendorf centrifuge tube. One gram of DMSO-d6 was added, followed by 8 µL of DCl in D_2O to digest the MOF crystals. Once the crystals were fully digested, the solution became slightly yellow. NOTE: If excess acid is used, the peak corresponding to 4-pyrazolecarboxylic acid shifts downfield and can overlap with the doublet corresponding to adenine causing erroneous integrations. To avoid this, we have found that using the minimum amount of acid to achieve a slightly yellow solution is ideal. Excess acid results in bright yellow solutions and peak overlap.

Attenuated total reflectance Fourier-transform infrared spectrometry (ATR-FT-IR) was conducted using a PerkinElmer Spectrum Two spectrometer with diamond/ZnSe ATR accessory. All spectra were collected using a $LiTaO_3$ MIR detector over a range of 450 to 4000 cm^{-1}. All spectra were processed using Spectrum 10 software.

Powder X-ray diffraction (PXRD) patterns were collected using a Bruker AXS D8 Discover powder diffractometer at 40 kV, 40 mA for Cu Kα, (λ= 1.5406 Å) with a scan speed of 0.20 sec/step from 3.0 to 40º at a step size of 0.02º. The data were analyzed using the EVA program from the Bruker Powder Analysis Software package. The simulated powder patterns were calculated using Mercury based on single crystal diffraction data of corresponding MOFs.

N_2 gas adsorption isotherms were collected on a Micromeritics 3-flex gas adsorption analyzer. All samples used with this instrument were activated under the specified conditions on a Micromeritics SmartVacPrep under vacuum and heat. **CO_2 gas adsorption isotherms were collected on a Quantachrome Autosorb-1 instrument.** For all experiments, approximately 20-50 mg of washed and/or solvent-exchanged sample was added into a pre-weighed sample analysis tube. A liquid N_2 bath was used for the N_2 adsorption experiments at 77 K. A dry ice/acetone bath was used for the CO_2 adsorption experiment at 195 K. Ultra-high purity grade N_2 and CO_2 gas cylinders (99.999 %) were used in this study.

Thermogravimetric analyses (TGA) were performed using a TGA Q500 thermal analysis system. All TGA experiments were performed under a N_2 atmosphere from about 20 °C to 800 °C at a rate of 1 °C /min. Data were analyzed using the TA Universal Analysis software package.

X-ray photoelectron (XPS) spectra were obtained using an ESCALAB 250XI XPS with a monochromated, micro-focused Al Kα X-ray source (spot size = 600 μm) before and after sputtering. Survey and high-resolution spectra were collected with a pass energy of 150 and 50 eV and a step size of 1.0 eV and 0.1 eV, respectively. The MOF samples also underwent an Ar ion sputtering (500 eV, 10 seconds) process to remove surface contaminants. Spectra were charge referenced to adventitious carbon (284.8 eV). Dry MOF samples were deposited onto p-doped (boron) silicon wafers (University Wafer, Boston, MA) that had been cleaned for ultra-high vacuum analysis.

Electron paramagnetic resonance (EPR) experiments were performed on a Bruker Elexsys E680 CW/FT X-band spectrometer using a Bruker ER4118X-MD5 resonator at 80 K. MOFs stored in DMF (<1mg) were aliquoted into a quartz sample tube of O.D. 4mm, I.D. 3mm. Continuous wave (CW)-EPR measurements were run with a center field of 3100 G, sweep width of 2000 G, modulation amplitude of 4 G, and a modulation frequency of 100 kHz for 1024 data points using a conversion time of 20.48 ms. All EPR simulations were performed using EasySpin software.[1]

Scanning electron microscopy (SEM) was conducted on a ZEISS Sigma500 VP operated at 1kV and was used to determine the crystal morphology and thickness of **bMOF-200** and **bMOF-201** membranes. For cross-sectional observation of membranes, alumina-supported membranes were broken into pieces and samples were mounted on 90 degrees specimen mount.

Elemental microanalyses were performed by the University of Illinois Urbana-Champaign Microanalysis Laboratory with an Exeter Analytical CE440. Inductively coupled plasma optical emission spectroscopy (ICP-OES) was used for metal concentration determination.

2. Synthesis and Characterization of as-synthesized bMOF-200

2.1 Synthesis of **as-synthesized bMOF-200**

The following stock solutions were prepared in DMF: 0.1 M $Zn(NO_3)_2{\cdot}6H_2O$, 0.1 M $ZnCl_2$ (anhydrous), 0.1 M $CuCl_2$ (anhydrous), 0.05 M adenine, 0.1 M H_2-pyz , and 0.1 M acetic acid. Stock solutions of adenine were heated and sonicated until fully dissolved. The reagents were added to a reaction tube with one end flame-sealed in the following order: $CuCl_2$ (200 μL), $Zn(NO_3)_2$ (300 μL), $ZnCl_2$ (300 μL), adenine (250 μL), H_2-pyz (200 μL), acetic acid (200 μL), and nanopure water (275 μL). The mixture became dark blue after addition of adenine, and then returned to a lighter shade of blue upon addition of water. The reaction vessel was then connected to a rubber hose with a stop cock adapter, frozen in liquid nitrogen for three minutes, then evacuated to approximately 100 mbar. The vessels were then flame sealed, allowed to thaw at room temperature, then placed in a 105 °C oven for 48 hours. After cooling to room temperature, slightly yellow cubic crystals were collected with a Pasteur pipette, placed in a 20 mL glass scintillation vial and washed with fresh, dry DMF (3x, 10 mL). Upon washing, a color change was observed from slight yellow to green. After three days, the crystals were dried under a stream of N_2 gas and further dried under vacuum using a Schlenk line for two hours. This product is notated as **as-synthesized bMOF-200**. Yield: 70% Anal. Calcd. (%): C, 32.15; H, 4.29; N, 20.45; Zn, 15.91; Cu, 7.73. Found: C, 32.12; H, 3.57; N, 19.72; Zn, 15.92; Cu, 7.26. Molecular Formula: $Zn_2Cu(4\text{-pyz})_2(ad)(DMF) \bullet (DMF)_2,(H_2O)_3$ (pyz = pyrazolate; ad = adeninate). Chemical Formula: $C_{22}H_{35}N_{12}O_{10}Zn_2Cu$ FT-IR (4000-450cm^{-1}): 3072 (br), 2930 (w), 2887 (br), 1674 (m), 1646 (s), 1622 (m), 1572 (m), 1537 (m), 1434 (m), 1413 (m), 1383 (m), 1281 (s), 1213 (m), 1092 (m), 1044 (m), 1005 (m), 798 (s).

Determination of Composition: Elemental analysis was used to determine the abundance of C, H, N, and Zn in a dried sample of **as-synthesized bMOF-200**. The data revealed a molecular formula of $Zn_2Cu(4\text{-pyz})_2(ad)(DMF) \bullet (DMF)_2,(H_2O)_3$ which closely matched the ratios of ligands, metals, and solvents observed in the refined crystal structure. ^{1}H-NMR was conducted to confirm the relative amounts of the organic components and any solvent within the framework (**Figure S1**). The resulting spectra yielded a 4-pyz : adenine ratio of approximately 2 : 1 and three DMF molecules, as well as some water. TGA analysis was then used to determine the mass percent of solvents (DMF and water) in the dried material by measuring the change in mass with increasing temperature (**Figure S2**). The solvent molecules (3 water and 3 DMF) account for approximately 33% of the total mass. The TGA plot shows a 33% weight loss by 250°C, well above the boiling point of both compounds. We note that the small pore windows within **bMOF-200** may inhibit solvent loss. All three characterization methods agree well with the determined molecular formula. XPS was used to determine the oxidation state of the Cu in a MeOH exchanged sample (*vide infra*) of **bMOF-200** (**Figure S3**). The Cu spectrum was collected before and after a 10 second sputtering from an Ar ion beam to etch the surface of the crystals. Before any etching occurred, the Cu spectrum shows Cu(II) character as evidenced by a satellite peak centred at 943 eV. The loss of this satellite peak and a concurrent shift in the $2p_{3/2}$ signal to lower binding energies in the post-etching spectrum indicate an increase in Cu (I) character, which suggests that surface oxidation occurs on bMOF-200 crystals, converting Cu (I) to Cu (II). We further investigated the Cu sites using EPR spectroscopy to gain insight into their coordination geometry (**Figure S4**). The spectrum for **as-synthesized bMOF-200** yielded an $A_{||}$ value

of 144 which is within the range of values assigned to a square planar geometry. Finally, PXRD patterns showed a nearly identical match to the simulated powder pattern derived from the single crystal structure, indicating the phase purity of the compound (**Figure S5**).

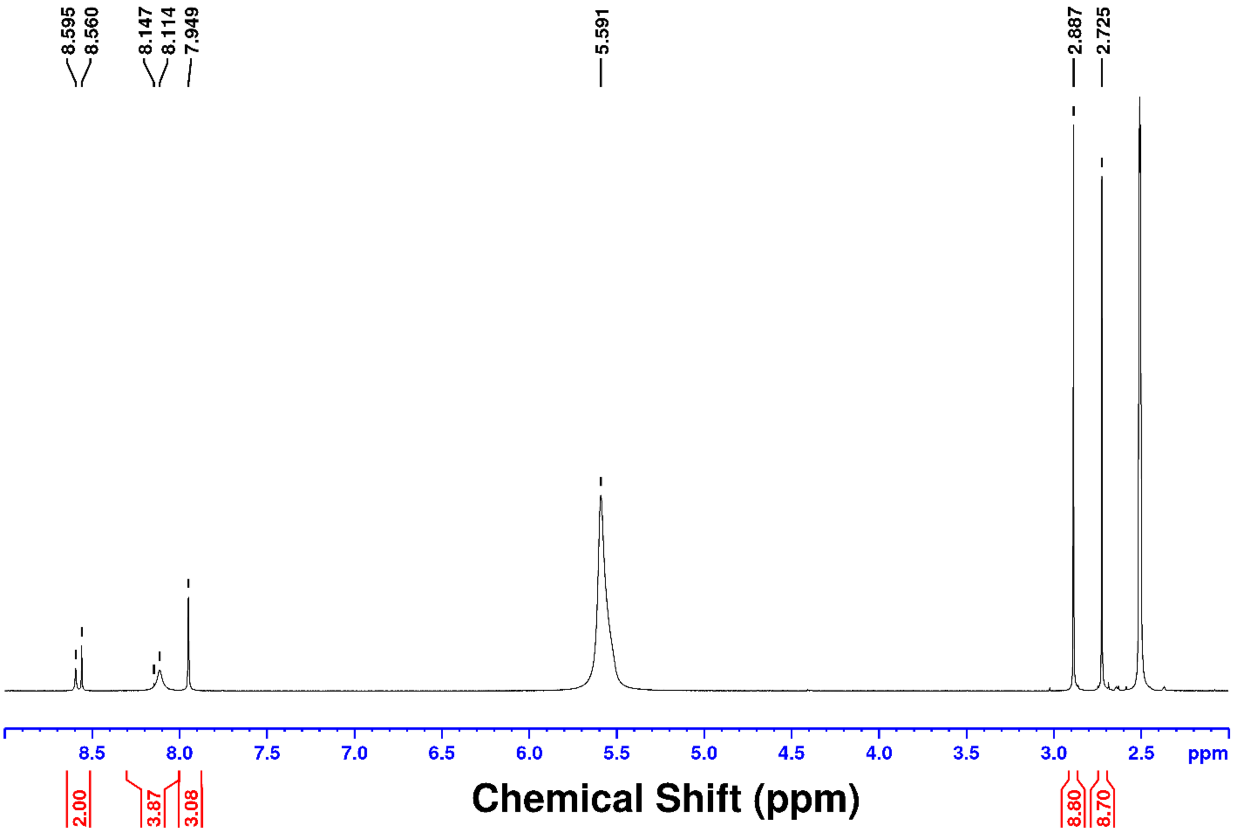

Figure S1. ^{1}H-NMR spectrum of **as-synthesized bMOF-200** after washing with DMF and drying on a Schlenk line for 1 hour. The peaks at 8.57 ppm corresponds to adenine protons. The broad peak at 8.114 ppm is assigned to 4-pyz protons. The small peak at 8.147 ppm is due to a small amount of formate from the decomposition of DMF. Peaks at 7.95, 2.89, and 2.73 ppm correspond to DMF. The integration shows approximately three DMF molecules per adenine molecule.

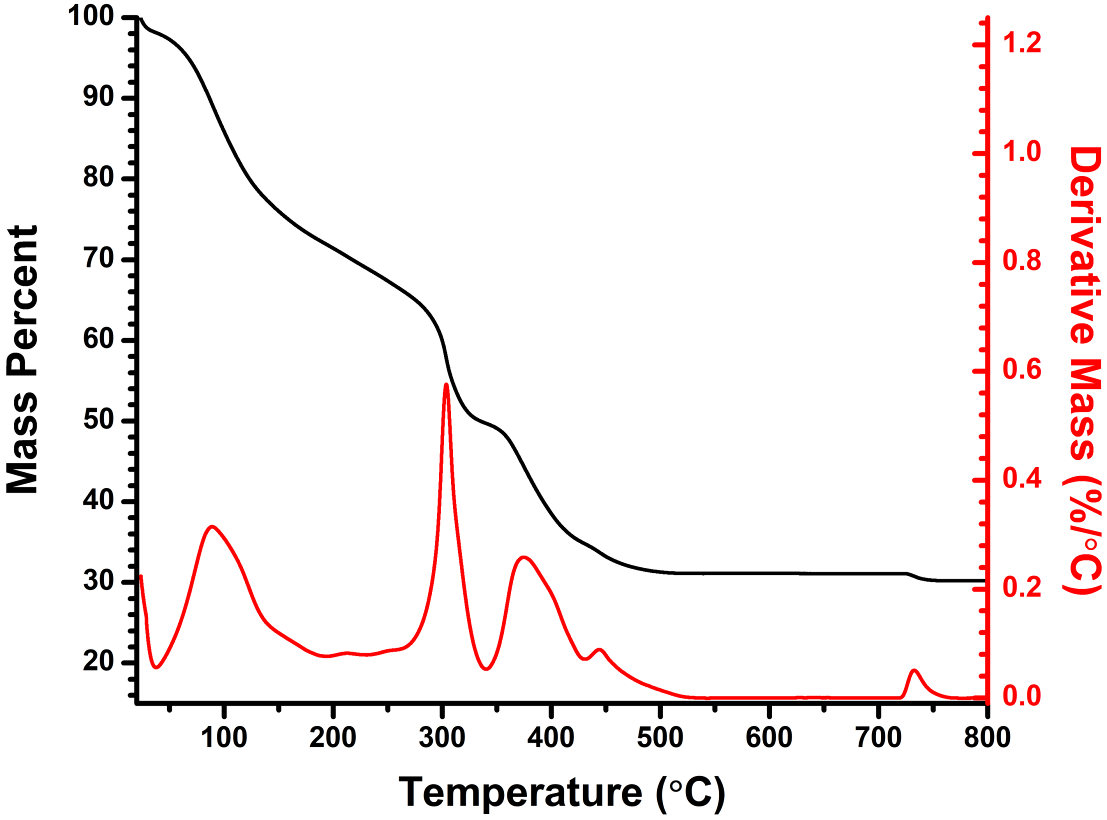

Figure S2. TGA of **as-synthesized bMOF-200**. The black curve shows the recorded weight loss with increasing temperature and the red curve is the first derivative weight loss with respect to temperature. ~33% of the sample weight is lost before 250 °C, which corresponds to 3 DMF and 3 H_2O. Subsequent weight loss steps are ascribed to sample decomposition.

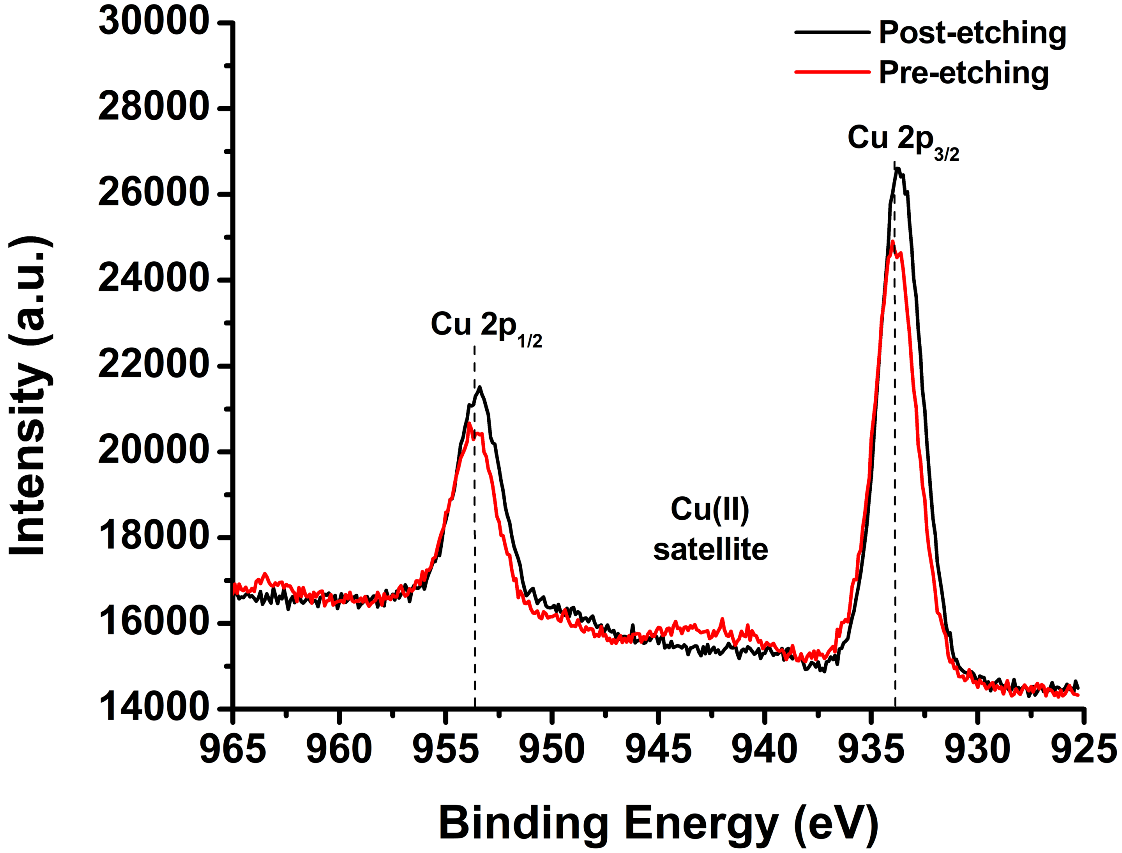

Figure S3. XPS of methanol-washed **bMOF-200** before (red) and after (black) surface etching with an Ar^+ beam. A slight shift in both Cu $2p_{1/2}$ and Cu $2p_{3/2}$ peaks to lower binding energies and the loss of the satellite peak between 945 and 940 eV indicate the presence of more Cu(I) character after etching. Therefore, we attribute the Cu(II) character primarily to surface oxidation of the MOF crystals in air.

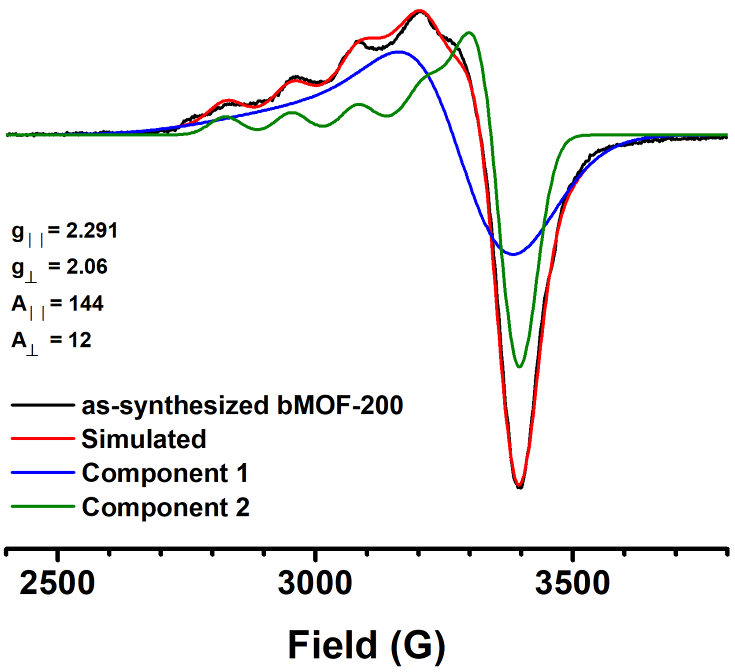

Figure S4. CW-EPR of **as-synthesized bMOF-200** with respective g and A tensor values. The simulated curve (red) is a combination of two separate simulated components: a broad, featureless signal (blue) and a signal with hyperfine splitting (green).

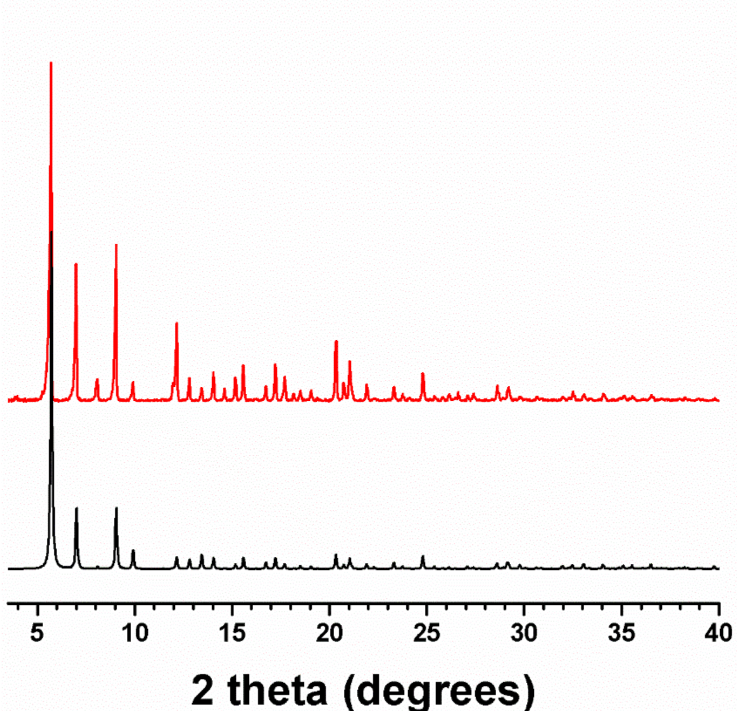

Figure S5. PXRD patterns of simulated (black) and **as-synthesized bMOF-200** (red). Simulated pattern was calculated from SC-XRD data.

2.2 Solvent exchange procedures

As-synthesized bMOF-200 crystals soaking in DMF were exchanged with 10 mL of dry methanol three times a day for three days. Then, the crystals were dried under a N_2 stream until they became a free-flowing powder. The composition of the dried samples was determined using a variety of characterization. The exchanged samples will be hereafter notated as **MeOH-bMOF-200**. Anal. Calcd. (%): C, 27.02; H, 3.33; N, 18.91; Zn, 19.62; Cu, 9.53. Found: C, 26.27; H, 1.56; N, 18.72; Zn, 19.30; Cu, 8.71. Molecular Formula: $Zn_2Cu(4\text{-pyz})_2(ad)(MeOH)$ • $(MeOH),(H_2O)_3$. Chemical Formula: $C_{15}H_{22}N_9O_9Zn_2Cu$ FT-IR (4000-450cm^{-1}): 3304 (br), 2982 (w), 2829 (w), 1677 (m), 1620 (w), 1554 (m), 1539 (m), 1283 (s), 1216 (m), 1047 (m), 1025 (m), 1007 (s), 895 (w), 819 (m), 798 (s).

Determination of Composition. After conducting solvent exchange procedures of **as-synthesized bMOF-200** with MeOH, the molecular formula of **MeOH-bMOF-200** was determined to be $Zn_2Cu(4\text{-pyz})_2(ad)(MeOH)$ • $(MeOH),(H_2O)_3$ by elemental analysis. ^{1}H-NMR of **MeOH-bMOF-200** showed complete removal of DMF without any loss of organic ligands 4-pyz and ad (**Figure S6**). The presence of two MeOH molecules and several water molecules were also observed. TGA analysis revealed an approximate loss 11% by mass by 230°C which correspond to three water molecules and one MeOH (calculated 12.9%) (**Figure S7**). The loss of the final MeOH molecule occurs at the small step between 230–280°C and accounts for an additional mass loss of ~5% (calculated 4.8%). We attribute this step to the loss of coordinated methanol, the displacement of which requires temperatures above the normal boiling point at atmospheric pressure. A

coordinated MeOH is indeed resolved in the single crystal structure of the methanol-exchanged crystal (see page S54). FT-IR spectroscopy was used to monitor the solvent exchange in bMOF-200 (**Figure S8**). The signals at 3304 (O–H stretch), 2829 (CH_3 stretch), 1385 (C–H in-plane rocking) and 1025 cm^{-1} (C–O stretch) correspond to the presence of MeOH and only appear in the **MeOH-bMOF-200** spectrum. Meanwhile, the signals at 2925 (CH_3 stretch), 1646 (carbonyl stretch), and 1092 (C–O stretch) cm^{-1} indicate the presence of DMF are only present in the **as-synthesized bMOF-200** spectrum, consistent with complete exchange of DMF with MeOH. PXRD confirmed the phase purity of **MeOH-bMOF-200** (**Figure S9**).

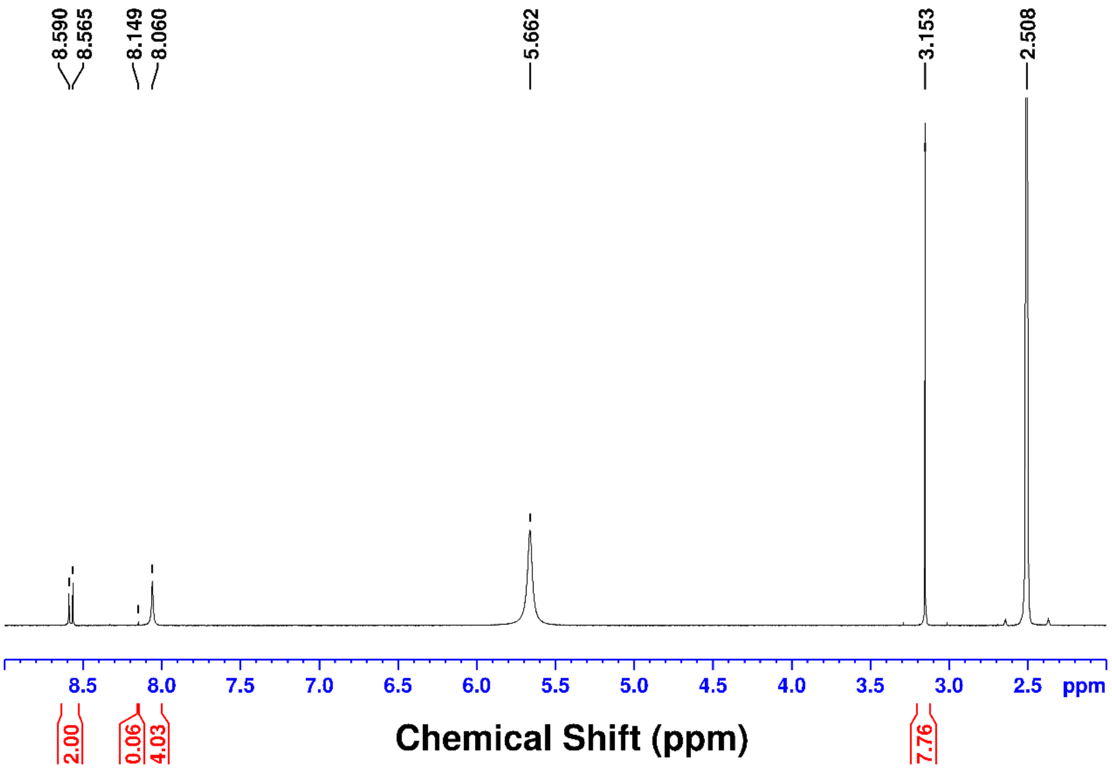

Figure S6. NMR spectrum of **MeOH-bMOF-200**. The disappearance of peaks at 7.95, 2.89, and 2.73 ppm and the emergence of a peak at 3.15 ppm correspond to complete replacement of DMF with MeOH. The integration shows approximately two molecules of MeOH for each adenine.

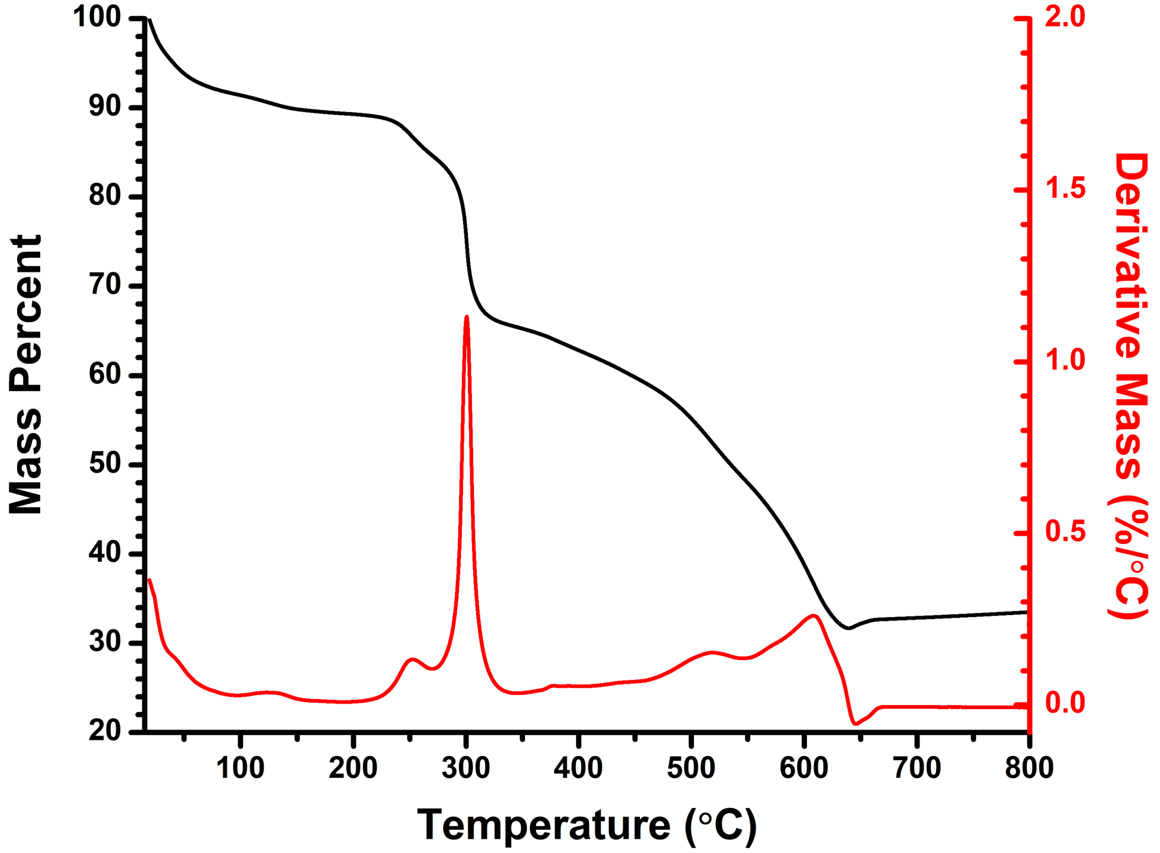

Figure S7. TGA of **MeOH-bMOF-200**. ~11% weight loss by 230 °C is attributed to uncoordinated MeOH and H_2O. Further loss of an additional 5% between 230°C and 287°C is attributed to the loss of coordinated MeOH. Further mass loss beyond 300°C is attributed to decomposition, consistent with the TGA collected on the as-synthesized MOF.

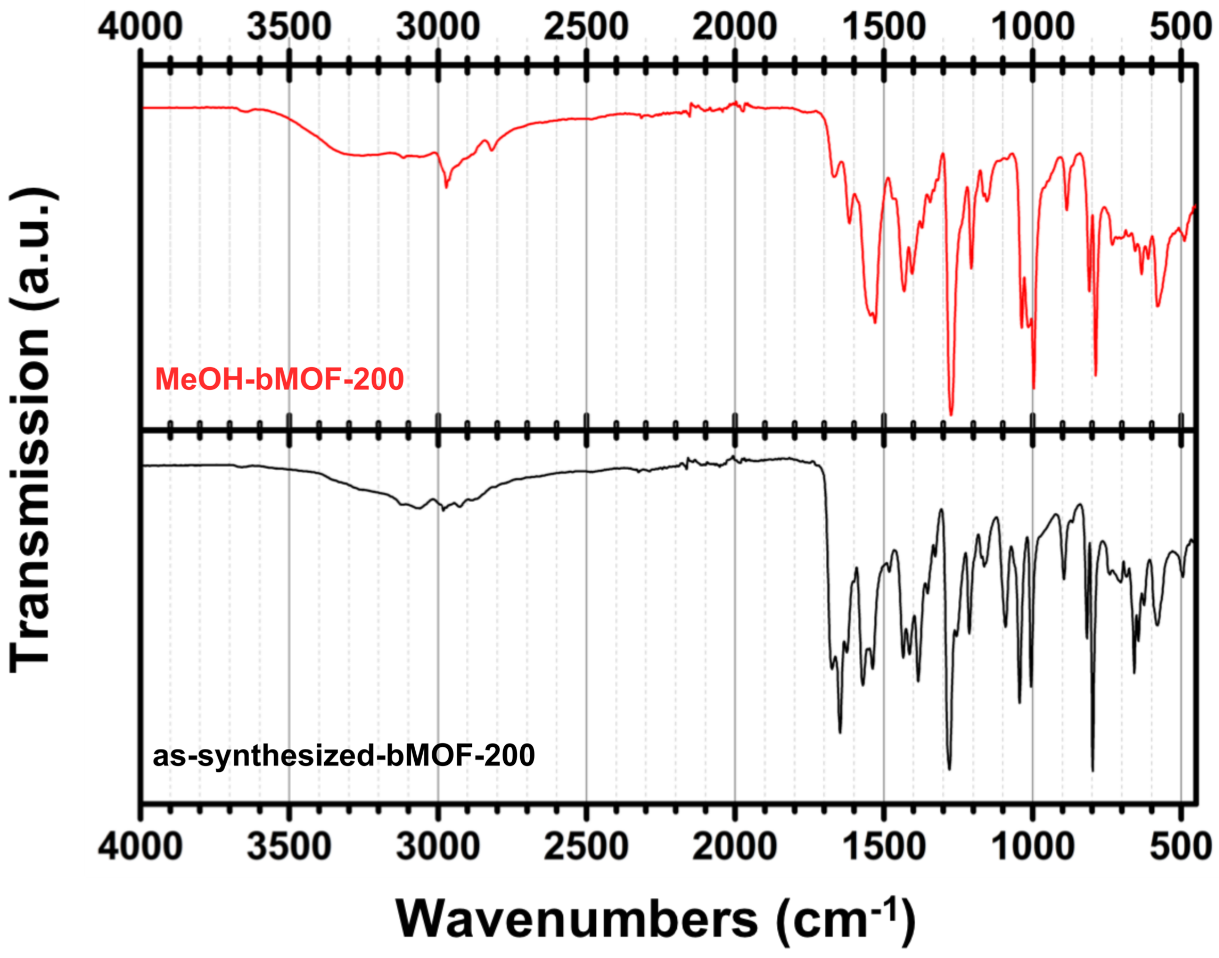

Figure S8. FT-IR spectra of **MeOH-bMOF-200** (red) and **as-synthesized bMOF-200** (black). The signals at 3304 (O–H stretch), 2829 (CH_3 stretch), 1385 (C–H in-plane rocking) and 1025 (C–O stretch) cm^{-1} correspond to the presence of MeOH while the peaks at 2925 (CH_3 stretch), 1646 (carbonyl stretch), and 1092 (C–O stretch) cm^{-1} indicate the presence of DMF.

A

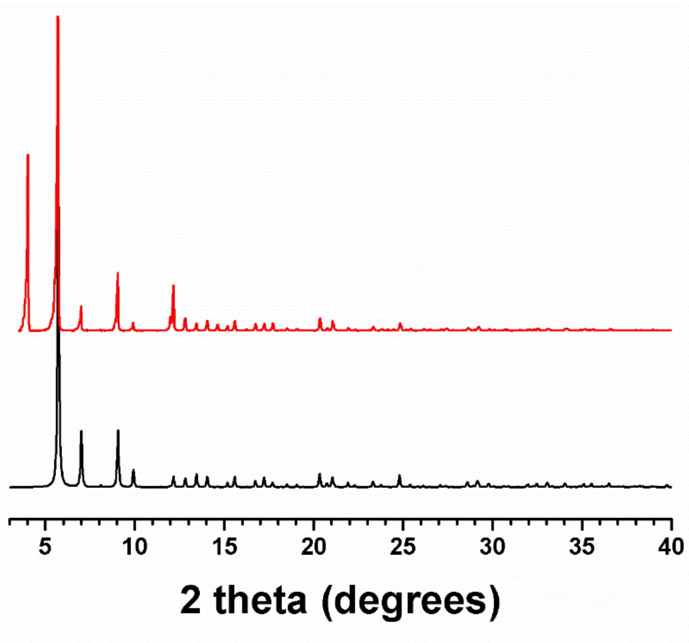

B

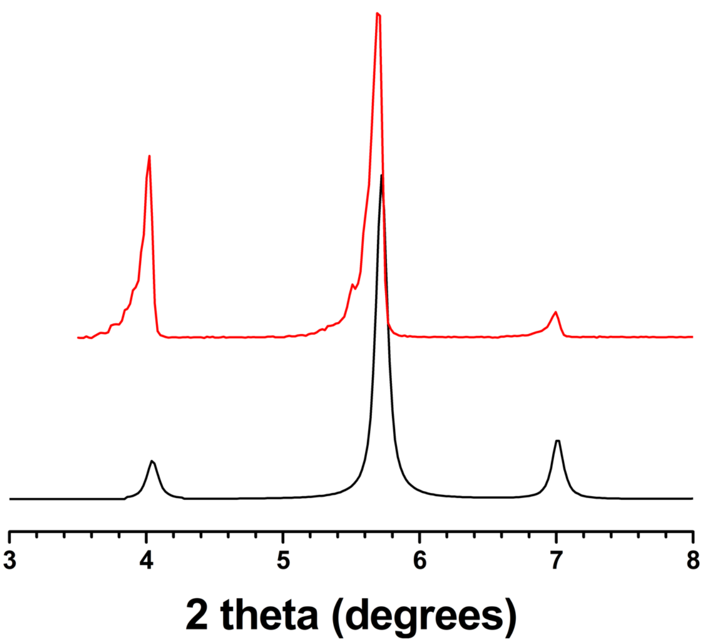

Figure S9. PXRD pattern of simulated (black) and **MeOH-bMOF-200** (red) (**A**). A peak at 4 2θ degrees becomes much more pronounced after solvent exchange with MeOH and matches a peak at the same position in the simulated PXRD pattern (**B**). The range between 3.8 and 4.2 2θ degrees in the simulated pattern in **Figure S9B** is multiplied by 100 to make this peak more apparent. This peak corresponds to the (200), which bisects the crystal along any of the equivalent faces and in which the coordinated DMF lies. The increase in intensity may result from replacing the coordinated DMF with MeOH.

2.3. Synthesis of **bMOF-201**

Unlike bMOF-200, **bMOF-201** syntheses did not require the use of evacuated tubes to obtain a pure product. In a typical reaction, 0.125 mmol (16.89 mg) adenine and 0.2 mmol (22.41 mg) H_2-pyz were added to a 20 mL scintillation vial followed by the addition of 0.02 mmol (0.4 mL) of 70% HBF_4. Then 4 mL of DMF were added to the mixture and the contents were dissolved using ultrasonication. Finally, 0.4 mmol (87.8 mg) of zinc acetate hexahydrate was added to the ligand solution. The resulting mixture was then sonicated again until all solids were dissolved. The vial was then capped with a PTFE lined cap and placed in a 120 °C oven for 16 hours. After removal from the oven, the resulting colourless cubic crystals were washed three times with 10 mL of fresh DMF. Solvent exchange with MeOH was performed on the DMF washed crystals by exchanging with dry MeOH (10mL) three times a day for three days. The crystals were then allowed to dry in air overnight and the resulting material was used for all characterization and analyses. Anal. Calcd. (%): C, 22.40; H, 4.00; N, 16.28; Zn, 24.48. Found: C, 22.72; H, 2.65; N, 16.26; Zn, 23.30. Molecular Formula: $Zn_{2.9}(pyz)_2(Adenine)(HCOO^-)_{0.45}(CH_3COO^-)_{0.35} \bullet (MeOH)_{0.3},(H_2O)_{10}$. Chemical Formula: $C_{14.45}H_{30.7}N_9O_{15.9}Zn_{2.9}$.

Determination of Composition: The composition of **bMOF-201** was determined by EA and yielded a molecular formula of $Zn_{2.9}(pyz)_2(Adenine)(HCOO^-)_{0.45}(CH_3COO^-)_{0.35} \bullet (MeOH)_{0.3},(H_2O)_{10}$. The ^{1}H-NMR spectra of **bMOF-201** revealed the expected pyz : adenine ratio of 2 : 1, as well as the presence of acetate, formate, and MeOH and water molecules (**Figure S10**). The monocarboxylate species acetate and formate are generated *in situ* during the solvothermal synthesis from the dissolution of $Zn(COOCH_3)_2$

and the decomposition of DMF, respectively. These species coordinate to the Zn at the Zn-ad motifs. The resultant Zn content of 2.9 in the molecular formula was determined based on the amount of charge balancing species determined by NMR and the Zn amount determined from the ICP elemental analysis results. We note that a Zn content of 3 was expected for the molecular formula, but there may be some missing Zn defects that account for the observed slight discrepancy. TGA analysis of **bMOF-201** revealed an initial loss of ~11% below 150 °C that corresponds to the MeOH and a portion of the water molecules within the framework (**Figure S11)**. The remaining water molecules are removed near at higher temperatures >290°C. We postulate that the removal of some solvent molecules via thermal heating requires elevated temperatures due to the blocking of P2 by coordinated monocarboxylates. A weight loss of ~5% at 200 °C is attributed to loss of coordinated formate and acetate (calculated 5.3%). Discrepancies between the elemental analysis, NMR, and TGA can be attributed to the hygroscopic nature of **bMOF-201**. The PXRD pattern matches very well to the simulated PXRD pattern generated from the single crystal structure, indicating phase purity (**Figure S12**).

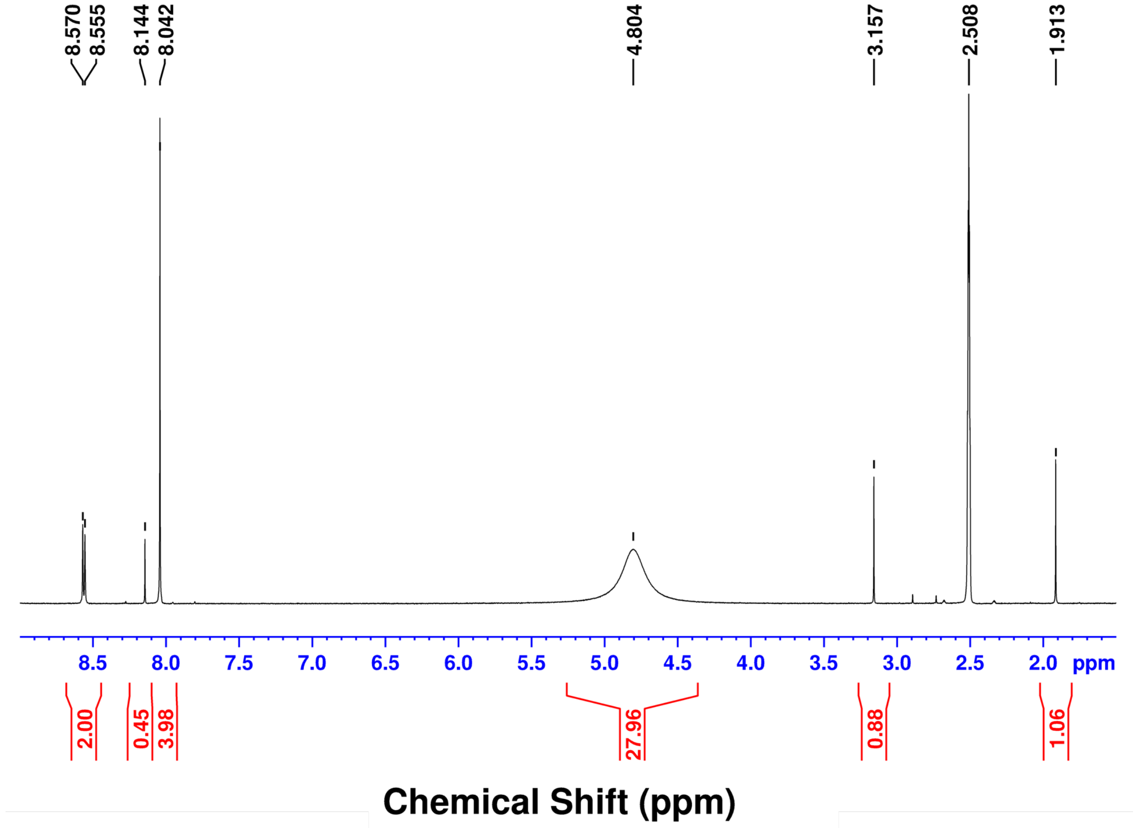

Figure S10. ^{1}H-NMR spectrum of **bMOF-201** after solvent exchange with MeOH and drying in air overnight. The signals due to adenine are at 8.57 ppm. The signal at 8.042 ppm corresponds to H_2-pyz protons. Charge-balancing coordinated ions formate and acetate are located at 8.14 ppm and 1.913 ppm, respectively. The signal at 3.15 ppm corresponds to MeOH. The integration shows approximately 0.45 formate, 0.35 acetate, and 0.3 MeOH molecules per adenine molecule.

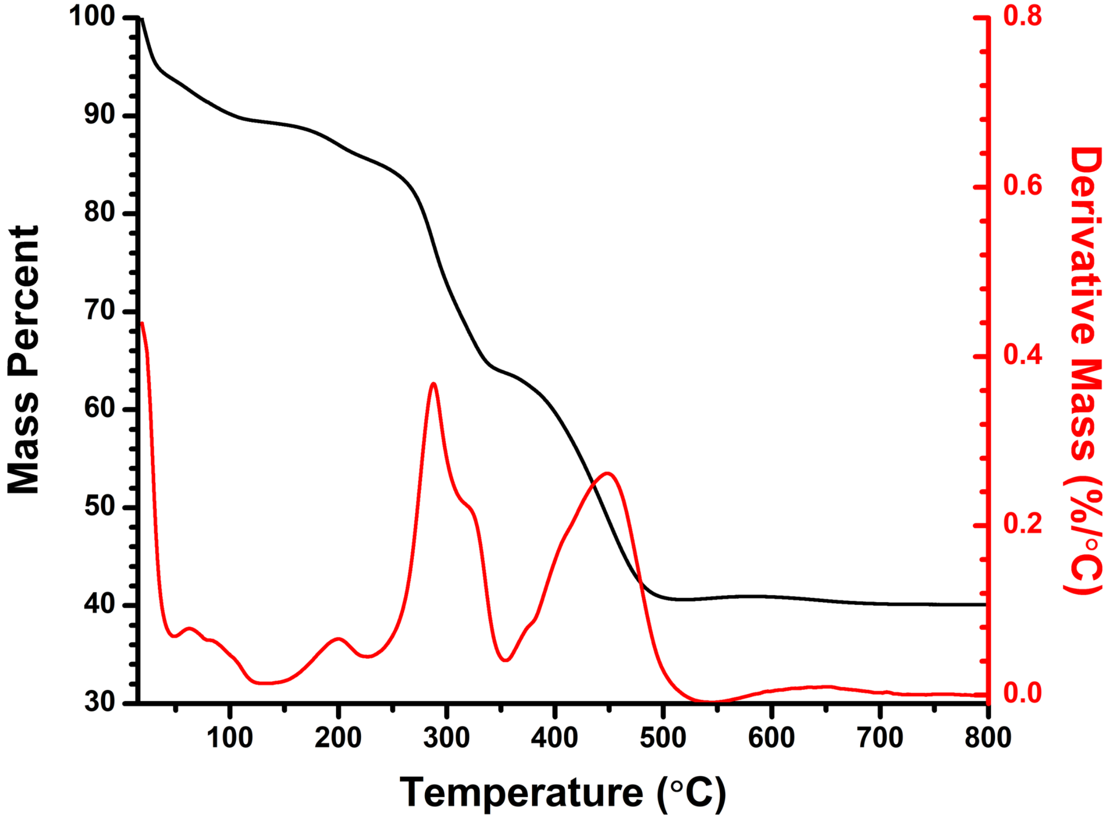

Figure S11. TGA of **bMOF-201**. The black curve shows the recorded weight loss with increasing temperature and the red curve is the first derivative weight loss with respect to temperature. The loss of a portion of the uncoordinated water molecules (~11%) and MeOH occurs before 100°C. The weight loss between 200°C and 250°C is attributed to the removal of coordinated monocarboxylates formate or acetate, which is approximately 5% of the total mass. Subsequent weight losses are attributed to framework decomposition and release of any trapped solvent.

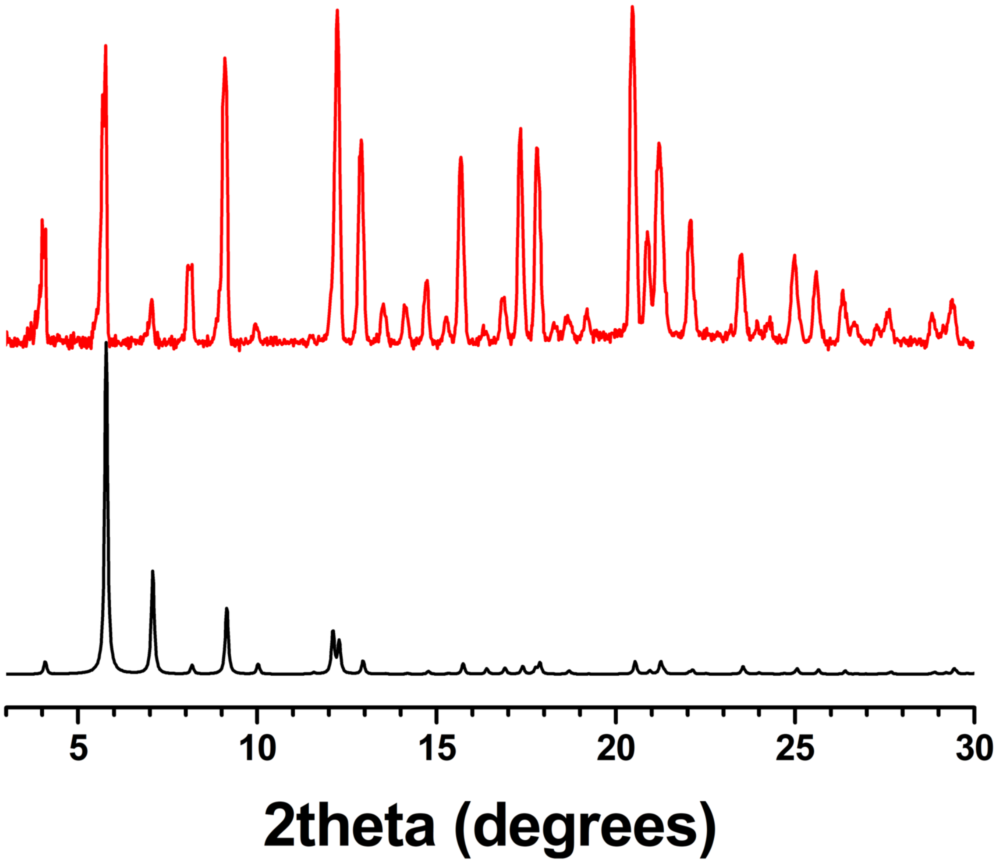

Figure S12. PXRD patterns of simulated (black) and synthesized **bMOF-201** (red). Simulated pattern was calculated from SC-XRD data.

3. Gas adsorption studies

3.1 Activation of **MeOH-bMOF-200**

MeOH-bMOF-200 was activated on a Micromeritics SmartVacPrep instrument. Approximately 50 mg of sample was added to a pre-weighed sample tube, which was installed onto an instrument port. The sample was then heated at 60°C for 1 hour, 100°C for 2 hours, then finally at 150°C for 24 hours under vacuum. For each temperature ramp, the rate was maintained at 10°C/min. After cooling back to room temperature, the sample was weighed again, then installed onto a Micromeritics 3-Flex gas adsorption analyzer.

3.2 Gas adsorption isotherms

The N_2 gas adsorption isotherm was collected on a Micromeritics 3-flex gas adsorption analyzer. The sample was activated at 150°C under 1.0 mmHg on a Micromeritics SmartVacPrep for 24 hours. A liquid N_2 bath was used for the N_2 adsorption experiments at 77 K. A Quantachrome Autosorb-1 instrument was used to activate the sample used for CO_2 adsorption. The material was held at 150°C under 1.0 mmHg for 24 hours. The CO_2 adsorption isotherm was collected on the same instrument using a dry ice/acetone bath at 195 K.

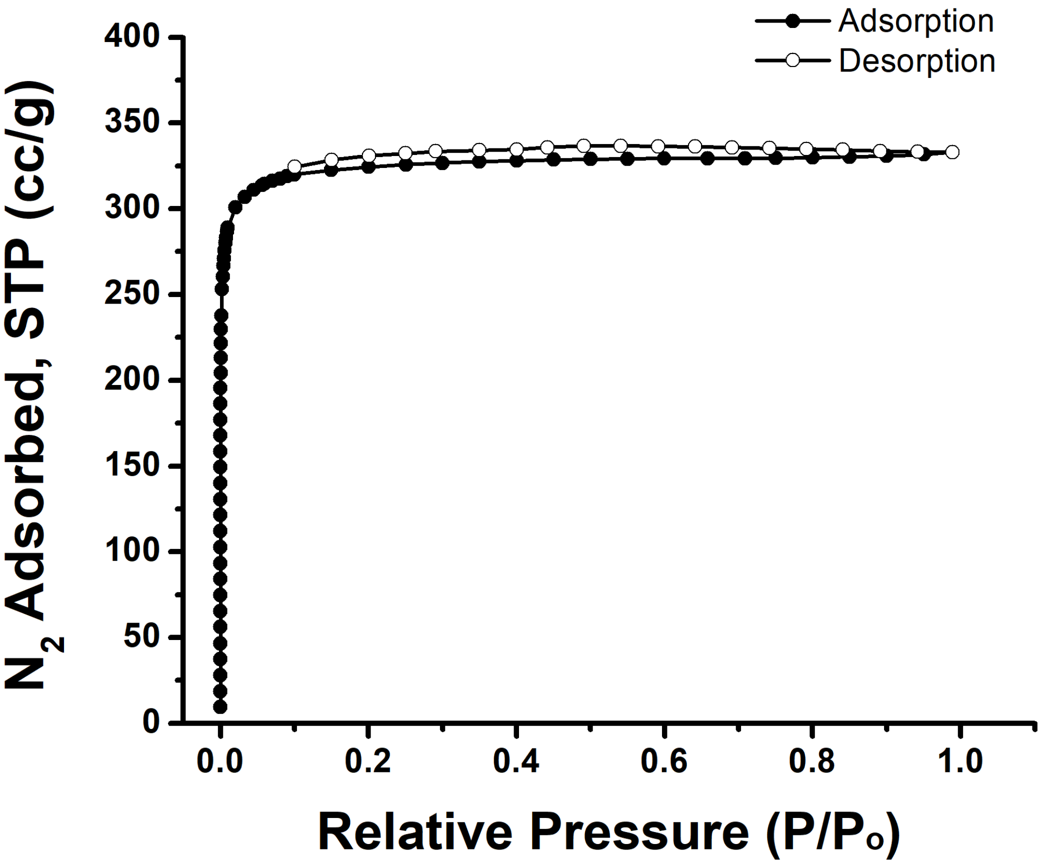

Figure S13. N_2 adsorption isotherm of **MeOH-bMOF-200** at 77 K after activation at 150°C under vacuum for 24 hrs. The calculated BET surface area was 1317 m^2g^{-1}.

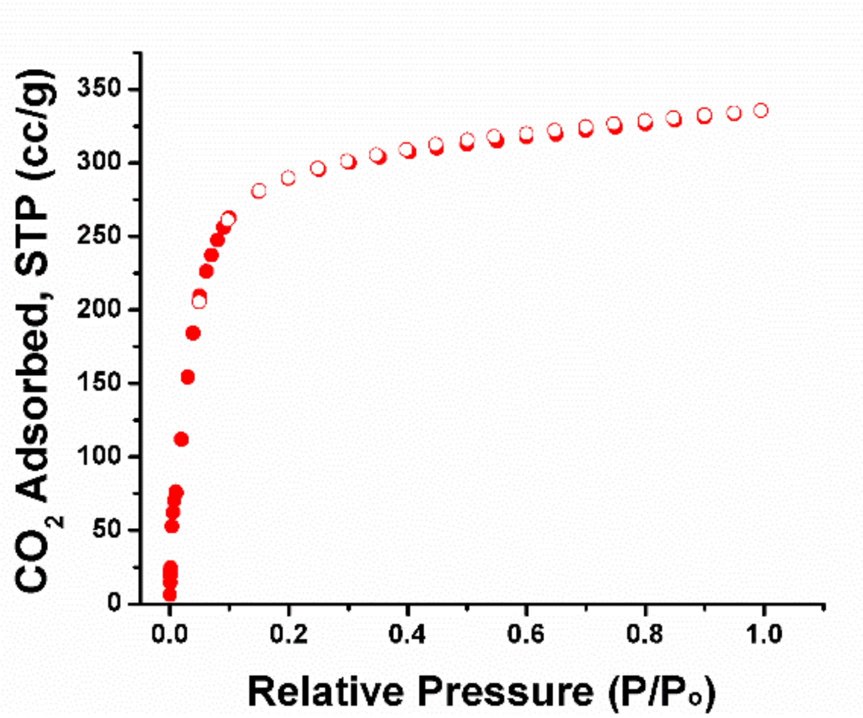

Figure S14. CO_2 adsorption isotherm of **MeOH-bMOF-200** collected at 195 K. The calculated BET surface area was 1741 m^2g^{-1}.

4. Computational methods

4.1 Computational Methodology

Density Functional Theory (DFT) calculations were used to examine physisorption of H_2 and CO_2 inside the MOF and the diffusion barrier of H_2 and CO_2 through the two distinctive openings on the Zn-pyrazolate cage. The structure of the MOF was obtained through SC-XRD. The base unit cell contains a total of 2448 atoms, which is computationally demanding, if not infeasible. Thus, we have generated a reduced unit cell with 888 atoms, by replacing some of the adenine ligands with their mirrored images. We believe this necessary simplification is valid, because one would expect stereochemistry at the substituted locations to play a minimum role in the adsorption and diffusion of linear molecules. In each physisorption calculation, we have placed one guest molecule inside the MOF and the entire structure was optimized with no geometric constraints. We have tested 15 unique initial placements in each set of H_2 and CO_2 physisorption calculations. The shortest distance between the adsorbate and the MOF at the optimized structure is reported in **Table S1**. The positioning of the CO_2 with the largest binding energy with bMOF-200 is shown in **Figure S15**.

Table S1. Physisorption energies and bond distances for either CO_2 or H_2 in the periodic **bMOF-200** in the reduced unit cell.

CO_2			H_2		
Physisorption energy (eV)	Bond length (Å)	Atoms (MOF-CO_2)	Physisorption energy (eV)	Bond length (Å)	Atoms (MOF-H_2)
-0.158	3.1762	Cu-O	-0.180	2.7587	Cu-H
-0.141	3.0727	Cu-O	-0.284	2.4542	H-H
-0.267	2.7073	H-O	-0.159	2.7646	Cu-H
-0.189	2.7755	H-O	-0.160	2.5529	H-H
-0.242	3.0794	C-O	-0.188	2.7997	O-H
-0.218	2.4798	H-O	-0.265	2.4696	H-H
-0.314	2.4330	H-O	-0.114	2.8810	N-H
-0.153	3.0904	C-C	-0.162	2.4993	O-H
-0.233	2.5631	H-O	-0.171	2.5761	H-H
-0.237	2.8552	H-O	-0.169	2.4880	O-H
-0.219	2.6039	H-O	-0.251	2.4898	H-H
-0.258	3.0871	O-C	-0.176	2.7841	O-H
-0.199	2.6361	H-O	-0.160	2.4887	O-H
-0.249	3.1634	Zn-O	-0.186	2.8867	Zn-H
-0.249	2.7536	H-O	-0.160	2.4893	O-H

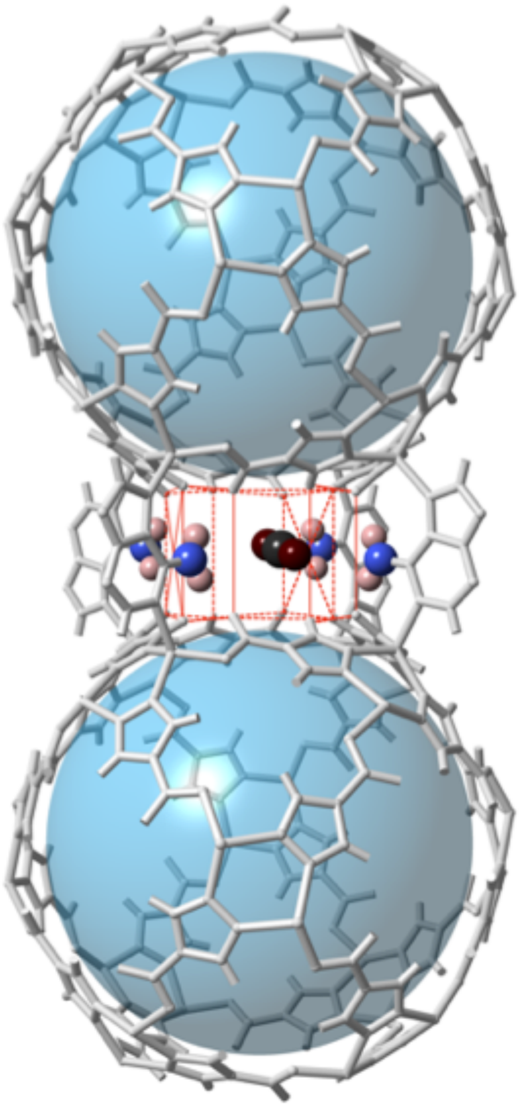

Figure S15. Periodic model of **bMOF-200** showing the position of CO_2 in P3, where it has its strongest binding energy as calculated from DFT. Primary amines are highlighted to emphasize their proximity to the adsorbed CO_2 molecule. Black, maroon, blue, and pink spheres indicate C, O, N, and H atoms, respectively. Red dashed lines highlight the pore boundaries of P3.

While the physisorption calculations were performed using the reduced unit cell, the diffusion barriers were predicted using a freestanding Zn-pyrazolate cage model. We have isolated the Zn-pyrazolate cage by terminating the dangling adeninate bonds with hydrogen atoms. This isolated cage retains both the square and triangular openings as

the cage inside the MOF. The difference in the neighboring H–H distance in the triangular and square opening is roughly 0.05 Å, and 0.1 Å, respectively, between the isolated cage and the periodic MOF at the optimized structures. We have calculated the minimum energy path for H_2 and CO_2 to travel from the center of the isolated cage to a point far (~6 Å) away from the cage through crossing each type of aperture. The reported distance in **Figure 3** is the distance between the center of mass of the adsorbate and the imaginary plane at the aperture along the optimized diffusion path. The plane for the triangular opening is defined by the positions of the hydrogen atoms. The plane for the square opening is defined by the vectors of the dihedral hydrogen atoms and their center of mass.

4.2 Density Functional Theory (DFT) Details

The DFT calculations performed here are done using the CP2K code.[2] The PBE functional was used to describe the exchange-correlations effects, and Grimme's D3 dispersion correction was included in order to describe van der Waals interactions inside the MOF.[3,4] Atomic species were described using the DZVP-MOLOPT basis set in combination with Geodecker, Teter and Hutter pseudopotentials, with a planewave cutoff of 360 Ry and relative cutoff of 60 Ry.[5] For the periodic system, the reduced unit cell has fixed angles of $\alpha = \beta = \gamma = 60°$, and the optimize unit cell length is 31.061 Å, which is in close agreements with the experimental value of 30.864 Å. The freestanding calculations were performed in a periodic 30 × 30 × 30 $Å^3$ cubic cell**.** The diffusion barrier of H_2 and CO_2 through the square and triangular openings on the freestanding Zn-pyrazolate cage was investigated using nudged elastic band method implemented in CP2K, and the force acting on any atom is below 0.077 eV/Å at the optimized geometry.[6,7] The DFT functional

used in this study has a mean absolute deviation (MAD) of 0.076 eV when benchmarked against the data set (S22x5) for interaction energies of noncovalently bonded complexes.[8,9] We believe the uncertainty in our calculated physisorption energy and diffusion barrier is similar in magnitude as the reported MAD. We do not expect systematic DFT errors, such as self-interaction, to be prominent for the weakly bound adsorbates investigated.[10]

5. Gas Breakthrough experiments

5.1 Breakthrough experiments

Breakthrough experiments were used to investigate the separation of H_2 and CO_2 by **bMOF-200**, **HKUST-1**, **ZIF-8**, and **UiO-66**. **HKUST-1**, **ZIF-8**, and **UiO-66** were solvent-exchanged and dried following gas adsorption activation procedures reported in the literature for these materials (see section 5.2 below). **bMOF-200** was solvent exchanged with MeOH, according to the preparation for gas adsorption studies and air dried prior to loading in the column (see Sections 2.2 and 3.1). MOF crystals were packed in a column with a diameter of 5 mm and a length of 50 mm and supported by quartz wool plugs on both ends of a quartz tube. Prior to the measurements the MOF-loaded column was activated *in situ* by continuous helium flow (20 sccm) at 150 °C (at 300°C for ZIF-8) with a ramp rate of 5 K/min for 12 hours. After activation, the column was cooled down to room temperature in helium flow. A mixture of 80 vol% H_2 and 20 vol % CO_2 was introduced to the column with a total flow rate of 5 sccm. The effluent gas was detected by mass spectrometry with a detection limit of 0.0008%. Any overshoot of H_2 above C/C_o = 1.0 in

the breakthrough experiments can be explained by the displacement of adsorbed H_2 by CO_2 via competitive adsorption.

5.2 Synthesis and characterization of reference MOFs

Synthesis of UiO-66

UiO-66 was synthesized following a literature procedure.[11] A ratio of H_2-BDC:Zr of 2:1 was used and the synthesis was performed at 200°C.

Synthesis of HKUST-1

HKUST-1 was synthesized following a literature procedure.[12]

Synthesis of ZIF-8

ZIF-8 was synthesized following a literature procedure.[13]

PXRD patterns were collected and compared to simulated patterns generated from the reported single crystal data in each respective reference (**Figure S16**). Gas adsorption isotherms for reference MOFs were collected on a Micromeritics 3-flex gas adsorption analyzer. All samples were activated under conditions specified in the cited literature on a Micromeritics SmartVacPrep under vacuum and heat. For all cases, approximately 50 mg of washed and/or solvent exchanged sample was added into a pre-weighed sample analysis tube. A liquid N_2 bath was used for N_2 adsorption experiments at 77 K. The BET surface areas for these synthesized MOFs are comparable to reported values (**Table S2**).

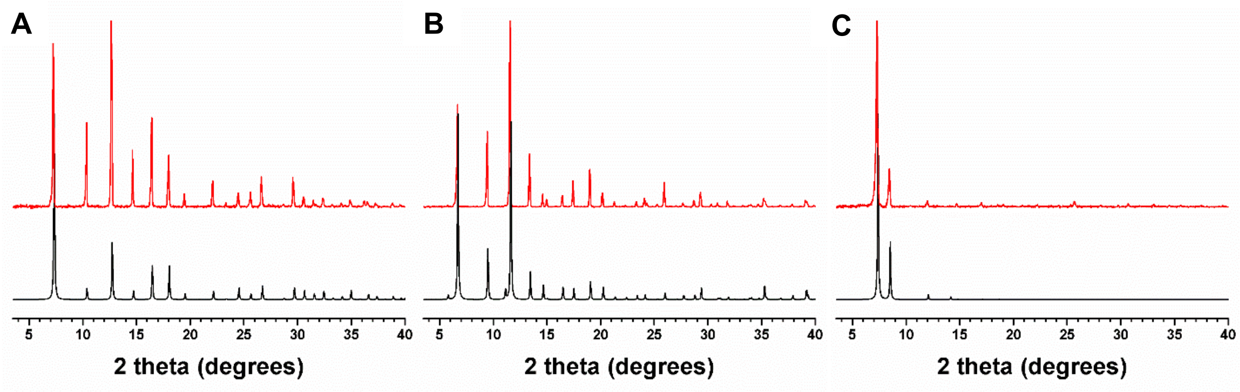

Figure S16. Experimental (red) and simulated PXRD patterns (black) of **HKUST-1** (A), **ZIF-8** (B), and **UiO-66** (C).

Table S2. Experimental BET surface area values for **HKUST-1**, **ZIF-8**, and **UiO-66** used in breakthrough experiments with corresponding literature values.

MOF	Experimental BET SA (m^2g^{-1})	Reported BET SA (m^2g^{-1})
**HKUST-1**	1419	1387
**ZIF-8**	1598	1630
**UIO-66**	1125	1212

6. Membrane fabrication and gas separation studies

6.1 Fabrication of **bMOF-200** Membranes

bMOF-200 membranes were synthesized on a homemade α-Al_2O_3 disk substrate *via* the secondary growth method. A 0.5 wt % **bMOF-200** crystal suspension was prepared in methanol. This suspension was used for dip coating the α-Al_2O_3 disk substrate, using a 30 s contact time. The seeded substrates were then placed horizontally in a glass reaction vessel and solvothermally treated with **bMOF-200** reaction components to form a **bMOF-200** layer. The molar composition of precursor solution for membrane synthesis was identical to that of **bMOF-200** crystal synthesis precursor solution. Repeated solvothermal growth up to five coats was employed in this study in order to form well-intergrown membrane. The as-synthesized membrane was washed repeatedly with dry DMF (3X) and immersed in dry methanol for two days for solvent exchange. Three membranes were synthesized under identical condition in order to verify reproducibility.

6.2 Fabrication of **bMOF-201** Membranes

A suspension of 0.5 wt % **bMOF-201** crystals was prepared in dry methanol for generating a seed layer on a homemade α-Al_2O_3 disk substrate. The seeded substrates prepared by dip coating method were immersed in precursor solution for **bMOF-201**. The molar composition of precursor solution was identical that used for **bMOF-201** crystal synthesis except for the amount of added HBF_4. To avoid seed crystal dissolution from the substrate due to acidic condition, the amount of HBF_4 was reduced by half. The reaction vessel was placed in a 120°C oil bath for 48 h. Repeated growth was not necessary for the **bMOF-201** membranes and well-intergrown membranes were successfully fabricated after

secondary growth. As-synthesized membranes were washed with dry DMF (3X) followed by solvent exchange with dry methanol. The solvent exchange procedure was identical to section 6.1. We confirmed the reproducibility by synthesizing three membranes under identical conditions from different batches.

6.3 Membrane Characterization

PXRD pattern of **bMOF-200** membranes (**Figure S17**) and **bMOF-201** membranes (**Figure S18**) showed a high degree of crystallinity with all matching peaks with powder XRD patterns of crystals. After repeated growth of **bMOF-200** membrane, the measured thickness was ~37.3 ± 4.3 μm, as determined from a cross-sectional view of membrane (**Figures 5a and 5b**). Rapid nucleation of **bMOF-201** crystals facilitated formation of well-intergrown membranes after the secondary growth, resulting in thicker membrane with thickness of ~62.7 ± 3.2 μm (**Figures 5c and 5d**). For both types of membranes, no visible cracks or defects were found on the surface. The H_2/CO_2 separation performances of **bMOF-200** and **bMOF-201** membranes are summarized in **Table S1** and **Table S2**. The permeance of each membrane was measured for three consecutive days and both permeances and separation factor remained unchanged. The points for bMOF-200 and bMOF-201 reported in the Robeson plot (**Figure 7**) are the average values of the three different membranes listed in **Tables S1** and **Table S2**.

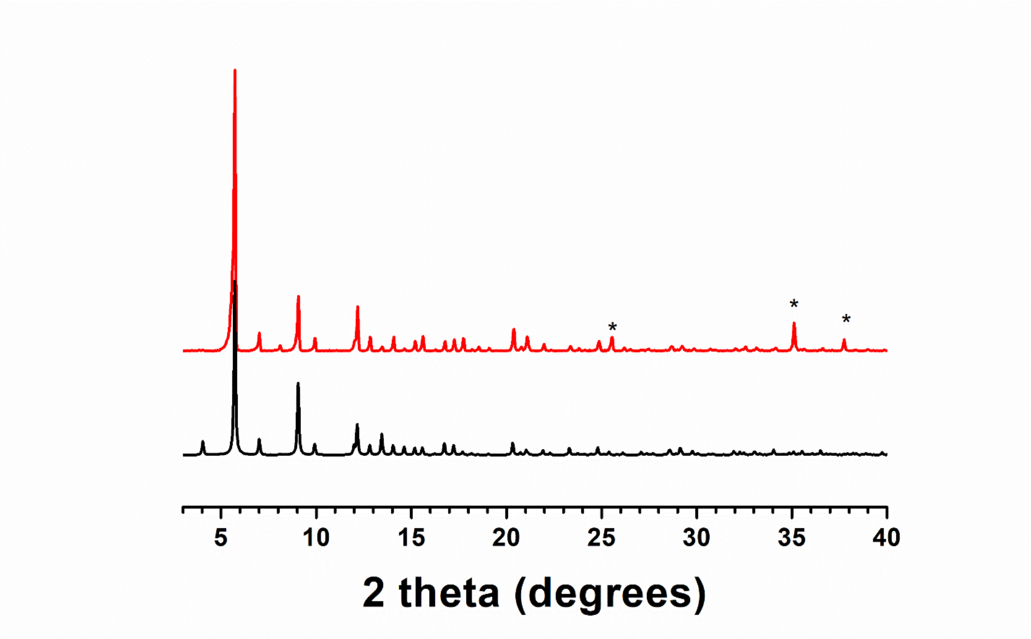

Figure S17. PXRD pattern of **as-synthesized bMOF-200** (black) and activated **bMOF-200** membrane prepared on α-Al_2O_3 substrate (red). Asterisks indicate α-Al_2O_3 diffraction lines.

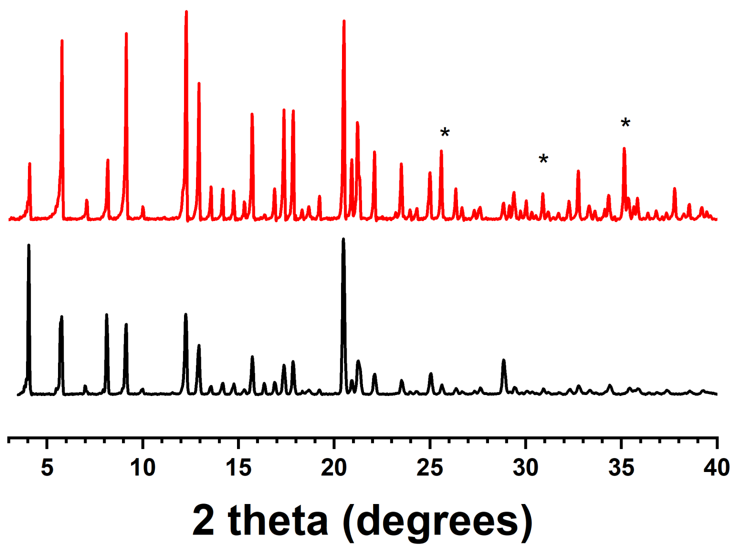

Figure S18. PXRD pattern of **bMOF-201** (black) and activated **bMOF-201** membrane prepared on α-Al_2O_3 substrate (red). Asterisks indicate α-Al_2O_3 diffraction lines.

6.4 Experimental Setup for Permeation Studies

After the solvent exchange, the membranes were activated at 423K for 24 hours in helium atmosphere before permeation measurement in order to remove any solvents molecules potentially remaining in the pore. For binary mixture permeation measurement, the membrane was sealed in a gas tight module with silicon o-rings. Membrane permeation behavior was investigated in Wicke-Kallenbach mode at a system pressure of 99 kPa for both feed and permeate side without pressure drop (**Scheme S1**). The feed containing a mixture of H_2 and CO_2 (50/50) was introduced to the membrane side with a total feed flow rate of 200 mL/min and the composition was adjusted using mass flow controller. The compositions of feed, permeate, and retentate were analyzed by a gas chromatograph (Agilent-GC8860). N_2 was introduced with volumetric flow rate of 50 mL/min to the permeate side as a sweep gas. The permeance of component *i* was calculated using the equation as follows

$$\overline{P_i} = \frac{J_i}{\Delta p_i}$$

where $\overline{P_i}$ is permeance (mol/m^2·s·Pa), J_i is steady-state flux (mol/m^2·s), and Δp_i is transmembrane partial pressure drop. Permeability (mol·m/m^2·s·Pa) was calculated as permeance multiplied by thickness of the membrane. The separation factor ($\alpha_{i,j}$) is defined as

$$\alpha_{i,j} = \frac{y_i/y_j}{x_i/x_j}$$

where y_i and y_j are mol fractions in permeate side, and x_i and x_j are molar composition in feed side for component *i* and *j*, respectively.

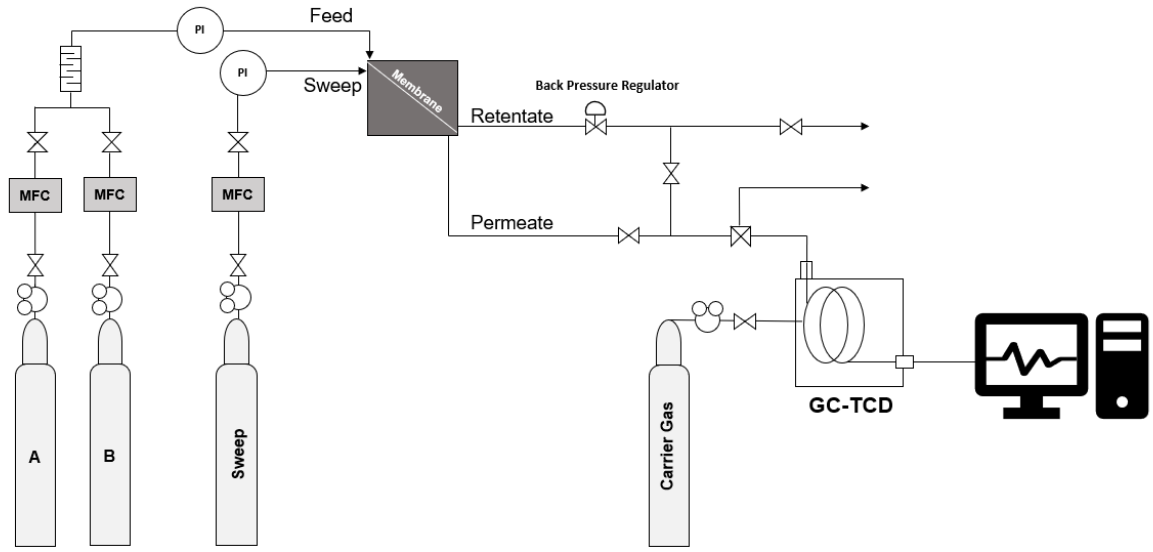

Scheme S1. Schematic diagram of binary mixture permeation system (A,B: target gas; MFC: mass flow controller; PI: pressure indicator).

Table S3. Separation performances of three **bMOF-200** membranes for the equimolar binary mixture of H_2/CO_2 at 25°C and 1 bar. The first column lists the iteration of the tested membrane, where M1 was the first membrane tested.

	Thickness (μm)	Permeance (mol / m² ·s·Pa)		Permeability (mol·m / m²·s·Pa)		Separation factor
		H_2	CO_2	H_2	CO_2	
M1	35.5	1.57×10^{-6}	1.24×10^{-7}	5.59×10^{-11}	4.40×10^{-12}	7.0
M2	43.3	1.26×10^{-6}	1.00×10^{-7}	5.48×10^{-11}	4.35×10^{-12}	6.9
M3	33.2	1.64×10^{-6}	1.10×10^{-7}	5.47×10^{-11}	3.67×10^{-12}	9.8

Table S4. Separation performances of three **bMOF-201** membranes for the equimolar binary mixture of H_2/CO_2 at 25°C and 1 bar. The first column lists the iteration of the tested membrane, where M4 was the first membrane tested.

	Thickness (μm)	Permeance (mol / m^2 ·s·Pa)		Permeability (mol·m / m^2·s·Pa)		Separation factor
		H_2	CO_2	H_2	CO_2	
M4	67.0	6.14 x 10^{-7}	2.17 x 10^{-8}	4.11 x 10^{-11}	1.45 x 10^{-12}	22.2
M5	59.5	6.55 x 10^{-7}	2.28 x 10^{-8}	3.90 x 10^{-11}	1.36 x 10^{-12}	20.0
M6	61.5	6.19 x 10^{-7}	1.94 x 10^{-8}	3.81 x 10^{-11}	1.19 x 10^{-12}	24.4

7. Single Crystal X-Ray Diffraction and Crystallographic Tables

7.1 **As-synthesized-bMOF-200**

Single crystal X-ray diffraction data for bMOF-200 was collected on a Bruker X8 Prospector Ultra diffractometer equipped with an Apex II CCD detector and an IμS micro-focus CuK-α X-ray source (λ= 1.54178 Å). A purple cubic crystal of dimensions 0.07 x 0.07 x 0.07 mm^3 was mounted on a goniometer using MiTeGen MicroMesh tips. Data was collected under N_2 stream at 230 K and processed using the Bruker APEX II software package.

A cubic unit cell with dimensions a = b = c = 43.6231(6) Å, α = β = γ = 90º, was derived from least squares refinement of 59240 reflections in range of 4.048º < 2θ < 107.766º. Centrosymmetric space group $Fm\bar{3}c$ was determined based on intensity statistics and systematic absences. The data were collected and integrated to 0.89 Å by Bruker program SAINT.[14] Empirical absorption correction was applied using program SADABS.[14] The structure was solved with direct method using SHELXT and refined by full-matrix least-squares on F^2 using SHELXL in Olex2.[15-17] All the non-H atoms were refined anisotropically. All the H atoms were refined isotropically. Crystallographic data are summarized in **Tables S5-S10**.

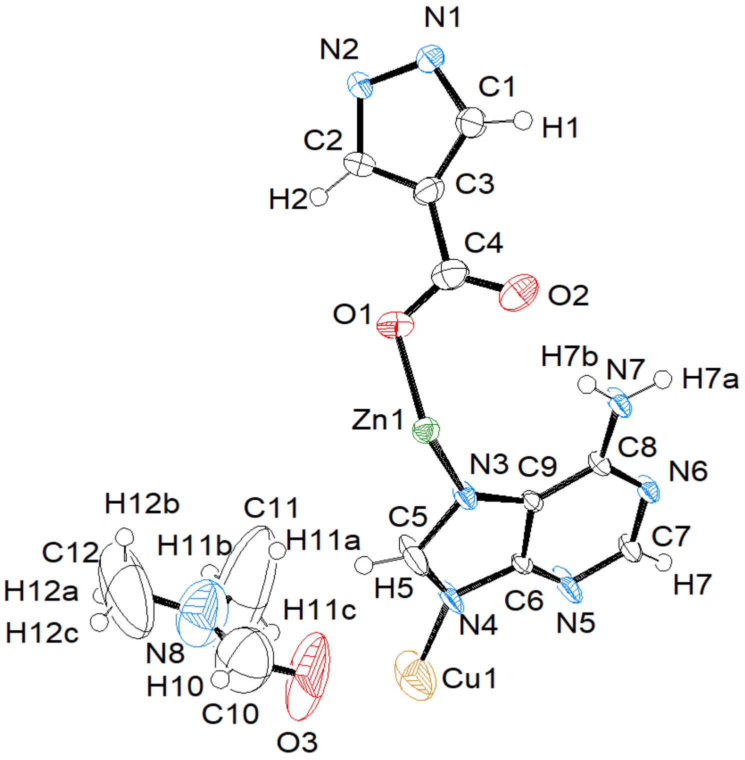

Figure S19. Asymmetric unit of **as-synthesized bMOF-200** with all non-H atoms represented by thermal ellipsoids drawn at 50% probability level produced by ORTEP-3[18] (O, red; C, black; N, blue; Zn, green; Cu, gold; H, black spheres).

Table S5. Crystallographic data and structural refinement for **as-synthesized bMOF-200**.

Identification code	**as-synthesized bMOF-200**
Empirical formula	$C_{15.5}H_{15}CuN_{10}O_5Zn_2$
Formula weight	615.65
Temperature/K	230.01
Crystal system	cubic
Space group	Fm-3c
a/Å	43.6231(6)
b/Å	43.6231(6)
c/Å	43.6231(6)
α/°	90
β/°	90
γ/°	90
Volume/Å^3	83014(3)
Z	96
ρ_{calc}g/cm^3	1.182
μ/mm^{-1}	2.625
F(000)	29472.0
Crystal size/mm^3	0.07 × 0.07 × 0.07
Radiation	CuKα (λ = 1.54178)
2Θ range for data collection/°	4.05 to 110.852
Index ranges	-33 ≤ h ≤ 45, -45 ≤ k ≤ 44, -44 ≤ l ≤ 42
Reflections collected	56571
Independent reflections	2312 [R_{int} = 0.0804, R_{sigma} = 0.0276]
Data/restraints/parameters	2312/78/188
Goodness-of-fit on F^2	1.131
Final R indexes [I>=2σ (I)]	R_1 = 0.0674, wR_2 = 0.2129
Final R indexes [all data]	R_1 = 0.0746, wR_2 = 0.2185
Largest diff. peak/hole / e Å^{-3}	2.89/-1.30

Table S6. Fractional atomic coordinates (x 10^4) and equivalent isotropic displacement parameters (Å^2 x 10^3) for **as-synthesized bMOF-200**. U_{eq} is defined as 1/3 of the trace of the orthogonalized U_{IJ} tensor.

Atom	*x*	*y*	*z*	U(eq)
Zn1	2800.0(2)	4202.1(2)	3790.7(2)	20.7(4)
Cu1	2881.5(6)	4670.1(4)	5000	73.4(8)
O1	3167.5(11)	4151.1(12)	3545.0(11)	40.7(13)
N1	3657.6(12)	4201.7(12)	2634.8(12)	25.7(13)
N2	3818.3(12)	4045.6(13)	2853.5(12)	25.6(13)
O2	2933.1(12)	4470.9(13)	3231.8(13)	51.5(15)
C3	3393.1(15)	4230.7(15)	3066.8(16)	29.1(16)
C2	3654.0(16)	4062.8(16)	3111.7(16)	32.3(17)
C1	3402.1(15)	4310.4(16)	2762.8(15)	30.5(16)
N4	2893(3)	4692(2)	4613.9(14)	36(3)
C5	2882(3)	4434.3(16)	4415.6(19)	47(5)
N3	2862(3)	4543.4(16)	4109.6(16)	23.0(14)
C9	2860(2)	4868.8(16)	4118.7(16)	15.5(18)
C6	2879(3)	4960.8(17)	4430.4(19)	15.5(18)
C4	3146.0(16)	4292.2(16)	3292.7(17)	34.0(17)
N8	3280(4)	3850(4)	5000	115(5)
O3	2861(4)	4149(3)	5000	164(8)
C10	2964(6)	3906(6)	5000	118(7)
C11	3456(7)	4118(6)	5000	330(40)
N6	2843(3)	5387(3)	4019(2)	23.0(14)
C8	2839(3)	5097(2)	3911(3)	18(2)
N7	2820(2)	5048(4)	3613.1(19)	26(3)
C7	2865(3)	5449(3)	4322(3)	23(3)
N5	2879(4)	5224(3)	4524(3)	42(4)
C12	3428(12)	3567(5)	5000	290(30)

Table S7. Anisotropic displacement parameters (Å^2 x 10^3) for **as-synthesized bMOF-200**. The anisotropic displacement factor exponent takes the form: $-2\pi^2[h^2a^{*2}U_{11}+2hka^*b^*U_{12}+\ldots]$.

**Atom**	**U_{11}**	**U_{22}**	**U_{33}**	**U_{23}**	**U_{13}**	**U_{12}**
Zn1	19.9(6)	20.5(6)	21.7(6)	0.1(3)	1.6(3)	0.0(3)
Cu1	131(2)	34.6(12)	54.6(13)	0	0	-4.6(11)
O1	43(3)	45(3)	35(3)	5(2)	18(2)	7(2)
N1	22(3)	29(3)	26(3)	2(2)	3(2)	4(2)
N2	29(3)	26(3)	22(3)	7(2)	3(2)	6(2)
O2	31(3)	65(4)	58(4)	6(3)	13(3)	17(3)
C3	26(4)	29(4)	32(4)	-1(3)	8(3)	4(3)
C2	39(4)	36(4)	22(4)	6(3)	11(3)	5(3)
C1	25(4)	39(4)	28(4)	4(3)	0(3)	6(3)
N4	72(10)	21(3)	13(3)	7(2)	-4(4)	1(3)
C5	104(14)	22(3)	17(3)	6(2)	-8(4)	3(4)
N3	34(4)	20(3)	15(3)	3.3(15)	-5(3)	0(2)
C9	15(5)	20(3)	12(3)	4.1(15)	-1(3)	0(2)
C6	15(5)	20(3)	12(3)	4.1(15)	-1(3)	0(2)
C4	33(4)	32(4)	37(4)	-5(3)	9(3)	-4(3)
N8	98(13)	74(11)	172(17)	0	0	0(9)
O3	159(15)	30(7)	300(20)	0	0	24(8)
C10	112(18)	95(17)	150(20)	0	0	-9(13)
C11	130(30)	79(19)	770(120)	0	0	-2(18)
N6	34(4)	20(3)	15(3)	3.3(15)	-5(3)	0(2)
C8	22(6)	20(3)	13(2)	3.7(16)	-3(3)	-1(2)
N7	45(5)	18(8)	14(2)	4(2)	-6(3)	-1(5)
C7	37(7)	18(3)	15(3)	2.7(19)	-5(4)	0(4)
N5	93(11)	21(3)	13(3)	4.0(19)	-7(4)	1(3)
C12	670(90)	60(15)	120(20)	0	0	70(30)

Table 8. Bond lengths for **as-synthesized bMOF-200**.

Atom	Atom	Length/Å	Atom	Atom	Length/Å
Zn1	O1	1.941(5)	N4	C5	1.4200
Zn1	N1[1]	1.983(5)	N4	C6	1.4200
Zn1	N2[2]	2.023(5)	C5	N3	1.4200
Zn1	N3	2.055(7)	N3	C9	1.4200
Cu1	Cu1[3]	2.878(4)	C9	C6	1.4200
Cu1	N4	1.688(6)	C9	C8	1.350(12)
Cu1	O3	2.274(13)	C6	N5	1.218(15)
O1	C4	1.265(9)	N8	C10	1.40(3)
N1	N2	1.366(8)	N8	C11	1.40(3)
N1	C1	1.334(9)	N8	C12	1.40(3)
N2	C2	1.337(8)	O3	C10	1.15(2)
O2	C4	1.241(9)	N6	C8	1.348(15)
C3	C2	1.367(10)	N6	C7	1.356(17)
C3	C1	1.372(10)	C8	N7	1.319(15)
C3	C4	1.485(9)	C7	N5	1.322(17)

[1]1/2-Z,+Y,+X; [2]+Z,+X,+Y; [3]+X,1-Y,+Z

Table S9. Bond angles for **as-synthesized bMOF-200**.

Atom	Atom	Atom	Angle/˚	Atom	Atom	Atom	Angle/˚
O1	Zn1	N1[1]	128.9(2)	N4	C5	N3	108.0
O1	Zn1	N2[2]	96.5(2)	C5	N3	Zn1	113.7(4)
O1	Zn1	N3	110.4(3)	C5	N3	C9	108.0
N1[1]	Zn1	N2[2]	105.7(2)	C9	N3	Zn1	137.9(4)
N1[1]	Zn1	N3	108.9(3)	N3	C9	C6	108.0
N2[2]	Zn1	N3	102.3(3)	C8	C9	N3	136.0(7)
N4	Cu1	Cu1[3]	86.7(3)	C8	C9	C6	116.0(7)
N4	Cu1	O3	93.3(3)	N4	C6	C9	108.0
O3	Cu1	Cu1[3]	177.8(4)	N5	C6	N4	125.9(7)
C4	O1	Zn1	111.3(4)	N5	C6	C9	126.1(7)
N2	N1	Zn1[4]	121.2(4)	O1	C4	C3	115.8(7)
C1	N1	Zn1[4]	130.1(4)	O2	C4	O1	123.2(6)
C1	N1	N2	108.3(5)	O2	C4	C3	121.0(7)
N1	N2	Zn1[5]	128.2(4)	C10	N8	C11	113(2)
C2	N2	Zn1[5]	124.9(4)	C12	N8	C10	128(3)
C2	N2	N1	106.6(5)	C12	N8	C11	119(3)
C2	C3	C1	104.5(6)	C10	O3	Cu1	154.7(18)
C2	C3	C4	127.3(7)	O3	C10	N8	123(2)
C1	C3	C4	128.1(6)	C8	N6	C7	122.0(11)
N2	C2	C3	110.8(6)	N6	C8	C9	117.1(10)
N1	C1	C3	109.8(6)	N7	C8	C9	123.1(10)
C5	N4	Cu1	124.2(6)	N7	C8	N6	119.8(11)
C5	N4	C6	108.0	N5	C7	N6	120.3(13)
C6	N4	Cu1	127.5(6)	C6	N5	C7	118.4(11)

[1]1/2-Z,+Y,+X; [2]+Z,+X,+Y; [3]+X,1-Y,+Z; [4]+Z,+Y,1/2-X; [5]+Y,+Z,+X

Table S10. Torsion angles for **as-synthesized bMOF-200.**

A	B	C	D	Angle/˚	A	B	C	D	Angle/˚
Zn1	O1	C4	O2	8.6(9)	N4	C6	N5	C7	180.0(11)
Zn1	O1	C4	C3	-171.0(5)	C5	N4	C6	C9	0.0
Zn1[1]	N1	N2	Zn1[2]	-0.4(7)	C5	N4	C6	N5	179.3(17)
Zn1[1]	N1	N2	C2	-173.5(4)	C5	N3	C9	C6	0.0
Zn1[1]	N1	C1	C3	173.6(5)	C5	N3	C9	C8	-178.6(13)
Zn1[2]	N2	C2	C3	-174.3(5)	N3	C9	C6	N4	0.0
Zn1	N3	C9	C6	171.9(11)	N3	C9	C6	N5	-179.3(17)
Zn1	N3	C9	C8	-6.8(18)	N3	C9	C8	N6	179.3(9)
Cu1[3]	Cu1	N4	C5	-176.6(7)	N3	C9	C8	N7	-1.5(19)
Cu1[3]	Cu1	N4	C6	-4.3(8)	C9	C6	N5	C7	-1(2)
Cu1	N4	C5	N3	173.6(10)	C6	N4	C5	N3	0.0
Cu1	N4	C6	C9	-173.3(10)	C6	C9	C8	N6	0.8(13)
Cu1	N4	C6	N5	6.0(15)	C6	C9	C8	N7	179.9(9)
Cu1	O3	C10	N8	0.0	C4	C3	C2	N2	177.5(6)
N1	N2	C2	C3	-1.0(8)	C4	C3	C1	N1	-177.3(7)
N2	N1	C1	C3	0.7(8)	O3	Cu1	N4	C5	1.1(9)
C2	C3	C1	N1	-1.2(8)	O3	Cu1	N4	C6	173.5(9)
C2	C3	C4	O1	-6.7(11)	C11	N8	C10	O3	0.0
C2	C3	C4	O2	173.6(7)	N6	C7	N5	C6	2(2)
C1	N1	N2	Zn1[2]	173.2(5)	C8	C9	C6	N4	178.9(10)
C1	N1	N2	C2	0.2(7)	C8	C9	C6	N5	-0.4(17)
C1	C3	C2	N2	1.4(8)	C8	N6	C7	N5	-1(2)
C1	C3	C4	O1	168.4(7)	C7	N6	C8	C9	0.0(17)
C1	C3	C4	O2	-11.2(12)	C7	N6	C8	N7	-179.2(12)
N4	C5	N3	Zn1	-174.1(8)	C12	N8	C10	O3	180.0
N4	C5	N3	C9	0.0					

[1]+Z,+Y,1/2-X; [2]+Y,+Z,+X; [3]+X,1-Y,+Z

Table S11. Hydrogen atom coordinates (Å x 10^4) and isotropic displacement parameters ($Å^2$x 10^3) for **as-synthesized bMOF-200**.

Atom	*x*	*y*	*z*	U(eq)
H2	3710.21	3971.85	3298.73	39
H1	3250.9	4424.61	2660.13	37
H5	2887.92	4227.62	4476.26	57
H10	2830.09	3737.91	5000	142
H11A	3454.03	4206.79	4796.1	489
H11B	3665.83	4074.19	5059.6	489
H11C	3366.53	4261.59	5144.3	489
H7A	2810.14	5202.35	3487.06	31
H7B	2818.03	4861.51	3542.64	31
H7	2871	5653.66	4389.58	28
H12A	3598.34	3572.52	5143.27	428
H12B	3504.9	3523.53	4795.82	428
H12C	3285.13	3408.3	5060.91	428

Table S12. Atomic occupancy for **as-synthesized bMOF-200**.

Atom	Occupancy	Atom	Occupancy	Atom	Occupancy
N4	0.5	C5	0.5	H5	0.5
N3	0.5	C9	0.5	C6	0.25
H11A	0.5	H11B	0.5	H11C	0.5
N6	0.5	C8	0.5	N7	0.5
H7A	0.5	H7B	0.5	C7	0.5
H7	0.5	N5	0.5	H12A	0.5
H12B	0.5	H12C	0.5		

7.2 **MeOH-bMOF-200**

Single crystal X-ray diffraction data for **MeOH-bMOF-200** was collected on a Bruker X8 Prospector Ultra diffractometer equipped with an Apex II CCD detector and an IμS micro-focus CuK-α X-ray source (λ= 1.54178 Å). A blue cubic crystal of dimensions 0.065 mm^3 was mounted on a goniometer using MiTeGen MicroMesh tips. Data was collected under N_2 stream at 150 K and processed using the Bruker APEX II software package.

A cubic unit cell with dimensions a = b = c = 43.571(5) Å, α = β = γ = 90º, was derived from least squares refinement of 59240 reflections in range of 4.048º < 2θ < 107.766º. Centrosymmetric space group $Fm\overline{3}c$ was determined based on intensity statistics and systematic absences. The data were collected and integrated to 0.89 Å by Bruker program SAINT.[14] Empirical absorption correction was applied using program SADABS.[14] The structure was solved with direct method using SHELXT and refined by full-matrix least-squares on F^2 using SHELXL in Olex2.[15-17] All the non-H atoms were refined anisotropically. All the H atoms were refined isotropically. The asymmetric unit is shown in **Figure S22**. Crystallographic data are summarized in **Tables S13-S20**.

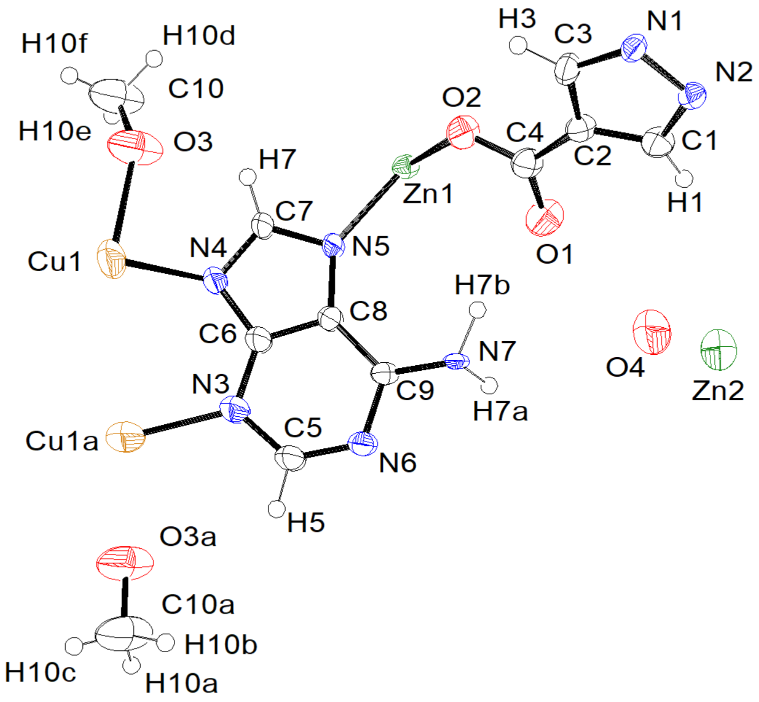

Figure S20. Asymmetric unit of **MeOH-bMOF-200** with all non-H atoms represented by thermal ellipsoids drawn at 50% probability level produced by ORTEP-3[18] (O, red; C, black; N, blue; Zn, green; Cu, gold; H, black spheres). In this structure, one Cu and one coordinating methanol are shown displaced over two positions, though the overall occupancy is still 0.5, which is consistent with the as-synthesized crystal structure.

Table S13. Crystal data and structure refinement for **MeOH-bMOF-200**.

Identification code	**MeOH-bMOF-200**
Empirical formula	$C_{13.75}H_{10.25}CuN_9O_{5.5}Zn_{2.08}$
Formula weight	589.29
Temperature/K	150
Crystal system	cubic
Space group	Fm-3c
a/Å	43.571(5)
b/Å	43.571(5)
c/Å	43.571(5)
α/°	90
β/°	90
γ/°	90
Volume/Å^3	82717(28)
Z	96
ρ_{calc}g/cm^3	1.136
μ/mm^{-1}	2.675
F(000)	27960.0
Crystal size/mm^3	0.08 × 0.08 × 0.08
Radiation	CuKα (λ = 1.54178)
2Θ range for data collection/°	4.056 to 102.648
Index ranges	-44 ≤ h ≤ 44, -44 ≤ k ≤ 44, -32 ≤ l ≤ 43
Reflections collected	169200
Independent reflections	1993 [R_{int} = 0.0850, R_{sigma} = 0.0155]
Data/restraints/parameters	1993/174/207
Goodness-of-fit on F^2	1.159
Final R indexes [I>=2σ (I)]	R_1 = 0.0461, wR_2 = 0.1415
Final R indexes [all data]	R_1 = 0.0505, wR_2 = 0.1456
Largest diff. peak/hole / e Å^{-3}	0.80/-0.54

Table S14. Fractional Atomic Coordinates (×104) and Equivalent Isotropic Displacement Parameters ($Å^2×10^3$) for **MeOH-bMOF-200**. Ueq is defined as 1/3 of the trace of the orthogonalised UIJ tensor.

Atom	x	y	z	U(eq)
Zn1	6202.0(2)	5797.0(2)	2798.8(2)	22.4(3)
Cu1	5000	4670.0(3)	2912.2(6)	71.6(6)
O2	6456.9(8)	5834.0(7)	3162.6(7)	38.1(9)
N1	7142.4(8)	5953.3(8)	3820.2(8)	22.5(9)
O1	6779.1(8)	5511.9(8)	2935.3(8)	46.3(10)
N2	7367.1(8)	5796.7(8)	3658.9(8)	23.7(9)
C2	6935.0(11)	5762.9(10)	3393.3(10)	26.9(11)
C3	6886.4(10)	5932.9(11)	3655.0(10)	28.8(12)
C1	7239.0(11)	5684.6(11)	3406.2(11)	29.4(12)
C4	6714.0(11)	5693.6(11)	3147.6(11)	31.4(12)
N3	5979(2)	4607.5(18)	2850(3)	30(2)
C5	5680(2)	4557(3)	2878(3)	39(2)
N4	5457(2)	4766.8(19)	2901(3)	52(2)
C6	5561.9(19)	5056.3(15)	2896.4(19)	42.3(18)
C7	5864(2)	5141.0(17)	2860(2)	28.3(16)
C8	6081(2)	4899.5(15)	2837.9(18)	22.9(17)
N5	6380.0(16)	4944(4)	2813.1(17)	28(5)
N9	5886(2)	5451.0(17)	2861(3)	26(2)
C9	5596(2)	5540(3)	2909(3)	39(2)
N11	5391(2)	5312.1(19)	2919(3)	50(2)
Zn2	7500	5000	2500	50.8(16)
O4	7194.7(18)	5000	2957(2)	82(3)
Cu1A	5000	5355.7(14)	3219(3)	78(3)
O3	5000	4141(4)	2974(8)	89(5)
C10A	5000	3853(5)	3030(30)	89(5)
C10	5000	6106(4)	3026(16)	89(5)
O3A	5089.81	5852(3)	3122(5)	89(5)

Table S15. Anisotropic Displacement Parameters (Å2×103) for **MeOH-bMOF-200**. The Anisotropic displacement factor exponent takes the form: -2π2[h2a*2U11+2hka*b*U12+…].

Atom	U_{11}	U_{22}	U_{33}	U_{23}	U_{13}	U_{12}
Zn1	26.7(5)	19.7(4)	20.8(5)	0.4(2)	-3.1(2)	2.3(2)
Cu1	51.6(11)	29.6(10)	133.7(19)	-3.8(11)	0	0
O2	38(2)	41(2)	36(2)	-4.9(15)	-15.3(16)	7.0(17)
N1	20(2)	24(2)	23(2)	-4.1(17)	0.0(17)	5.1(16)
O1	51(2)	54(2)	34(2)	-14(2)	-13.0(18)	11.3(19)
N2	22(2)	24(2)	25(2)	-2.7(17)	0.0(18)	2.3(16)
C2	32(3)	25(3)	23(3)	-1(2)	-4(2)	4(2)
C3	20(3)	35(3)	31(3)	-4(2)	-3(2)	5(2)
C1	34(3)	34(3)	21(3)	-6(2)	1(2)	3(2)
C4	34(3)	29(3)	31(3)	2(2)	-6(2)	5(2)
N3	30(4)	20(3)	40(5)	-3(3)	-10(4)	-1(2)
C5	31(4)	20(4)	66(6)	3(4)	-10(4)	-2(2)
N4	29(4)	22(3)	105(6)	4(4)	-5(4)	-2(2)
C6	23(3)	21(3)	83(5)	2(3)	-7(3)	-0.3(18)
C7	22(3)	20(2)	43(4)	2(3)	-10(3)	1.0(17)
C8	27(3)	18(3)	23(4)	-2(3)	-11(3)	1.3(17)
N5	26(4)	18(15)	40(4)	-5(4)	10(3)	-5(4)
N9	17(3)	19(2)	40(5)	5(3)	-11(4)	2(2)
C9	18(4)	21(3)	79(6)	1(4)	-7(4)	1(2)
N11	20(3)	22(3)	107(6)	1(4)	-5(4)	-1(2)
Zn2	46(2)	61(4)	46(2)	0	0	0
O4	49(5)	66(6)	131(8)	0	-24(5)	0
Cu1A	22(3)	42(4)	169(9)	12(5)	0	0
O3	80(8)	36(5)	150(12)	-4(8)	-84(11)	11(7)
C10A	80(8)	36(5)	150(12)	-4(8)	-84(11)	11(7)
C10	80(8)	36(5)	150(12)	-4(8)	-84(11)	11(7)
O3A	80(8)	36(5)	150(12)	-4(8)	-84(11)	11(7)

Table S16. Bond Lengths for **MeOH-bMOF-200**.

Atom	Atom	Length/Å	Atom	Atom	Length/Å
Zn1	O2	1.942(3)	C5	N4	1.338(7)
Zn1	N1[1]	2.006(4)	N4	C6	1.342(7)
Zn1	N2[2]	1.976(4)	C6	C7	1.376(14)
Zn1	N9	2.059(9)	C6	N11	1.345(7)
Cu1	Cu1[3]	2.876(3)	C7	C8	1.419(11)
Cu1	N4	2.036(9)	C7	N9	1.354(7)
Cu1	Cu1A[3]	1.342(11)	C8	N5	1.320(12)
Cu1	O3	2.320(17)	N9	C9	1.340(7)
O2	C4	1.278(6)	C9	N11	1.338(7)
N1	N2	1.385(5)	N11	Cu1A	2.155(13)
N1	C3	1.330(6)	Zn2	O4[4]	2.396(10)
O1	C4	1.250(6)	Zn2	O4[5]	2.396(10)
N2	C1	1.328(6)	Zn2	O4[6]	2.396(10)
C2	C3	1.376(6)	Zn2	O4	2.396(10)
C2	C1	1.369(7)	Cu1A	O3A	2.237(14)
C2	C4	1.471(7)	O3	C10A	1.28(3)
N3	C5	1.330(7)	C10	O3A	1.25(3)
N3	C8	1.349(7)			

[1]+Y,1-Z,1-X; [2]1-Z,+Y,-1/2+X; [3]1-X,1-Y,+Z; [4]3/2-X,1-Y,1/2-Z; [5]1/2+Z,+Y,1-X; [6]1-Z,1-Y,-1/2+X

Table S17. Bond Angles for **MeOH-bMOF-200**

Atom	Atom	Atom	Angle/˚	Atom	Atom	Atom	Angle/˚
O2	Zn1	N1^{1}	97.80(14)	C5	N4	Cu1	124.7(6)
O2	Zn1	N2^{2}	126.98(15)	C5	N4	C6	113.2(8)
O2	Zn1	N9	109.6(4)	C6	N4	Cu1	121.9(7)
N1^{1}	Zn1	N9	103.3(2)	N4	C6	C7	125.4(7)
N2^{2}	Zn1	N1^{1}	106.73(15)	N4	C6	N11	126.1(8)
N2^{2}	Zn1	N9	109.3(4)	N11	C6	C7	108.5(7)
N4	Cu1	Cu1^{3}	78.0(3)	C6	C7	C8	116.6(7)
N4	Cu1	O3	102.0(3)	N9	C7	C6	109.6(8)
Cu1A^{3}	Cu1	Cu1^{3}	94.8(3)	N9	C7	C8	133.8(9)
Cu1A^{3}	Cu1	N4	92.3(4)	N3	C8	C7	118.5(8)
Cu1A^{3}	Cu1	O3	78.5(9)	N5	C8	N3	117.9(11)
O3	Cu1	Cu1^{3}	173.3(8)	N5	C8	C7	123.6(10)
C4	O2	Zn1	114.8(3)	C7	N9	Zn1	141.0(7)
N2	N1	Zn1^{4}	126.7(3)	C9	N9	Zn1	116.0(6)
C3	N1	Zn1^{4}	126.4(3)	C9	N9	C7	102.9(8)
C3	N1	N2	106.6(3)	N11	C9	N9	114.8(9)
N1	N2	Zn1^{5}	121.1(3)	C6	N11	Cu1A	123.8(7)
C1	N2	Zn1^{5}	130.9(3)	C9	N11	C6	104.1(8)
C1	N2	N1	107.8(3)	C9	N11	Cu1A	118.8(8)
C3	C2	C4	127.8(4)	O4^{6}	Zn2	O4^{7}	90.000(1)
C1	C2	C3	104.4(4)	O4	Zn2	O4^{7}	180.0
C1	C2	C4	127.7(4)	O4^{5}	Zn2	O4^{7}	90.000(2)
N1	C3	C2	110.8(4)	O4^{5}	Zn2	O4	90.0
N2	C1	C2	110.4(4)	O4^{6}	Zn2	O4^{5}	180.0(3)
O2	C4	C2	116.0(4)	O4^{6}	Zn2	O4	90.004(2)
O1	C4	O2	122.7(4)	Cu1^{3}	Cu1A	N11	52.3(4)
O1	C4	C2	121.3(4)	Cu1^{3}	Cu1A	O3A	83.8(7)
C5	N3	C8	118.9(9)	C10A	O3	Cu1	177(7)
N3	C5	N4	127.3(9)	C10	O3A	Cu1A	150.1(9)

^{1}+Y,1-Z,1-X; ^{2}1-Z,+Y,-1/2+X; ^{3}1-X,1-Y,+Z; ^{4}1-Z,+X,1-Y; ^{5}1/2+Z,+Y,1-X; ^{6}1-Z,1-Y,-1/2+X; ^{7}3/2-X,1-Y,1/2-Z

Table S18. Hydrogen Atom Coordinates (Å × 10^4) and Isotropic Displacement Parameters ($Å^2$ × 10^3) for **MeOH-bMOF-200**.

Atom	x	y	z	U(eq)
H3	6695.86	6023.47	3710.14	35
H1	7343.53	5566.69	3255.5	35
H5	5616.79	4348.21	2881.74	46
H5A	6511.09	4872.43	2632.43	34
H5B	6479.5	5162.88	2803.6	34
H9	5539.74	5749.79	2933.46	47
H10A	5000	3739.51	2831.35	134
H10B	5183.6	3799.01	3143.85	134
H10C	4816.3	3799.01	3143.85	134
H10D	5164.9	6256.63	3049.52	134
H10E	4945.9	6087.13	2808.22	134
H10F	4819.7	6172.33	3142.52	134

Table S19. Atomic Occupancy for **MeOH-bMOF-200**.

Atom	Occupancy	Atom	Occupancy	Atom	Occupancy
Cu1	0.8	N3	0.5	C5	0.5
H5	0.5	N4	0.5	C6	0.5
C7	0.5	C8	0.5	N5	0.5
H5A	0.5	H5B	0.5	N9	0.5
C9	0.5	H9	0.5	N11	0.5
Zn2	0.3334	O4	0.75	Cu1A	0.2
O3	0.125	C10A	0.125	H10A	0.125
H10B	0.125	H10C	0.125	C10	0.25
H10D	0.25	H10E	0.25	H10F	0.25
O3A	0.25				

Table S20. Solvent masks information for **MeOH-bMOF-200**.

Number	X	Y	Z	Volume	Electron count	Content
1	0.000	0.000	0.000	2842.8	235.3	?
2	0.000	0.000	0.500	2842.8	235.3	?
3	0.000	0.500	0.000	2842.8	235.3	?
4	0.000	0.500	0.500	2842.8	235.3	?
5	0.250	0.250	0.250	2025.8	594.2	?
6	0.250	0.250	0.750	2025.8	608.7	?
7	0.250	0.750	0.250	2025.8	608.7	?
8	0.250	0.750	0.750	2025.8	594.2	?
9	0.500	0.000	0.500	2842.8	235.3	?
10	0.500	0.000	0.000	2842.8	235.3	?
11	0.500	0.500	0.500	2842.8	235.3	?
12	0.500	0.500	0.000	2842.8	235.3	?
13	0.750	0.250	0.250	2025.8	608.7	?
14	0.750	0.250	0.750	2025.8	594.2	?
15	0.750	0.750	0.250	2025.8	594.2	?
16	0.750	0.750	0.750	2025.8	608.7	?

7.3 **bMOF-201**

Single crystal X-ray diffraction data of **bMOF-201** were collected on a Bruker X8 Prospector Ultra diffractometer equipped with an Apex II CCD detector and an IµS micro-focus CuK-α X-ray source (λ= 1.54178 Å). A colorless cubic crystal of dimensions 0.065x0.065x0.065 mm^3 was mounted on a goniometer using MiTeGen MicroMesh tips. Data were collected under N_2 stream at 150 K and processed using the Bruker APEX II software package.

A cubic unit cell with dimensions a = b = c = 43.1097(5) Å, α = β = γ = 90º, was derived from least squares refinement of 9745 reflections in range of 5.798º < 2θ < 117.966º. Centrosymmetric space group $Fm\bar{3}c$ was determined based on intensity statistics and systematic absences. The data were collected and integrated to 0.90 Å by Bruker program SAINT.[14] Empirical absorption correction was applied using program SADABS.[14] The structure was solved with direct method using SHELXT and refined by full-matrix least-squares on F^2 using SHELXL in Olex2.[15-17] All the non-H atoms were refined anisotropically. All the H atoms were refined isotropically. Refinement details are included in the CIF. The asymmetric unit is shown in **Figure S23**. Crystallographic data are summarised in **Tables S21-S29**.

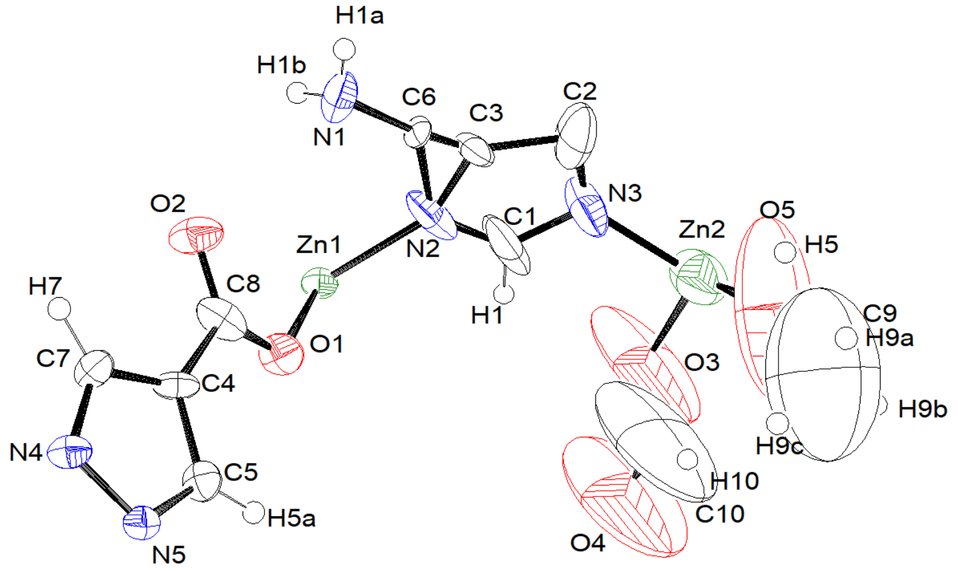

Figure S21. Asymmetric unit of **bMOF-201** with all non-H atoms represented by thermal ellipsoids drawn at 50% probability level produced by ORTEP-3[18] (O, red; C, black; N, blue; Zn, green; H, black spheres).

Table S21. Crystallographic data and structural refinement for **bMOF-201**.

Identification code	**bMOF-201**
Empirical formula	$C_{15}H_{13}N_9O_7Zn_3$
Formula weight	627.45
Temperature/K	150.0
Crystal system	cubic
Space group	Fm-3c
a/Å	43.1097(5)
b/Å	43.1097(5)
c/Å	43.1097(5)
α/°	90
β/°	90
γ/°	90
Volume/Å^3	80117(3)
Z	96
ρ_{calc}g/cm^3	1.248
μ/mm^{-1}	2.865
F(000)	29952.0
Crystal size/mm^3	0.65 × 0.65 × 0.65
Radiation	CuKα (λ = 1.54178)
2Θ range for data collection/°	4.1 to 117.928
Index ranges	-47 ≤ h ≤ 47, -47 ≤ k ≤ 37, -47 ≤ l ≤ 43
Reflections collected	79464
Independent reflections	2535 [R_{int} = 0.0507, R_{sigma} = 0.0132]
Data/restraints/parameters	2535/33/178
Goodness-of-fit on F^2	1.110
Final R indexes [I>=2σ (I)]	R_1 = 0.0814, wR_2 = 0.2406
Final R indexes [all data]	R_1 = 0.0850, wR_2 = 0.2445
Largest diff. peak/hole / e Å^{-3}	2.13/-1.48

Table S22. Fractional atomic coordinates (x 10^4) and equivalent isotropic displacement parameters ($Å^2$ x 10^3) for **bMOF-201**. U_{eq} is defined as 1/3 of the trace of the orthogonalized U_{IJ} tensor.

Atom	x	y	z	U(eq)
Zn1	2800.1(2)	5786.6(2)	3817.8(2)	25.3(4)
Zn2	3257.5(7)	5389.9(5)	5000	88.0(9)
O1	3157.0(12)	5810.8(13)	3546.6(12)	38.5(13)
O2	2932.3(13)	5484.3(14)	3221.6(14)	45.6(14)
O3	3176(10)	5852(3)	5000	400(20)
O4	3370(13)	6318(5)	5000	460(30)
O5	3688(4)	5190(3)	5000	360(30)
N1	2800(2)	5000	3653(2)	43(2)
N2	2872.6(18)	5428.2(18)	4113(2)	58(2)
N3	3017(2)	5273(2)	4611(2)	73(3)
N4	3663.9(13)	5777.4(13)	2628.7(14)	28.5(14)
N5	3822.5(13)	5940.8(13)	2860.4(13)	25.9(13)
C1	2950(3)	5495(3)	4399(3)	82(4)
C2	2980(4)	5000	4482(3)	73(5)
C3	2884(4)	5136(3)	4177(4)	35(4)
C4	3396.7(15)	5740.8(16)	3064.7(18)	29.0(16)
C5	3656.6(17)	5923.6(16)	3117.6(17)	30.2(16)
C6	2849(3)	5096(3)	3958(4)	23(3)
C7	3411.8(17)	5655.2(17)	2756.4(16)	30.3(16)
C8	3141.9(18)	5669.8(18)	3293(2)	38(2)
C9	4040(6)	5182(14)	5000	410(40)
C10	3404(10)	6022(7)	5000	450(30)

Table S23. Anisotropic displacement parameters ($Å^2 \times 10^3$) for **bMOF-201**. The anisotropic displacement factor exponent takes the form: $-2\pi^2[h^2a^{*2}U_{11}+2hka^*b^*U_{12}+\ldots]$.

Atom	U_{11}	U_{22}	U_{33}	U_{23}	U_{13}	U_{12}
Zn1	24.8(6)	21.5(6)	29.7(6)	-2.8(4)	3.1(4)	-0.2(4)
Zn2	138(2)	63.8(14)	62.3(14)	0	0	-17.4(14)
O1	34(3)	49(3)	33(3)	-4(3)	13(2)	-4(2)
O2	36(3)	47(3)	54(4)	-12(3)	10(3)	-14(3)
O3	1010(80)	54(7)	134(16)	0	0	-97(16)
O4	1030(90)	96(12)	260(30)	0	0	-140(30)
O5	500(60)	450(60)	119(17)	0	0	310(50)
N1	32(5)	69(7)	27(5)	0	-5(4)	0
N2	55(5)	43(4)	76(6)	36(4)	18(4)	9(4)
N3	101(7)	58(5)	61(6)	31(5)	-3(5)	12(5)
N4	24(3)	27(3)	35(3)	-3(2)	3(2)	-5(2)
N5	27(3)	24(3)	27(3)	0(2)	0(3)	-3(2)
C1	83(8)	86(9)	77(9)	62(7)	15(7)	1(7)
C2	75(11)	116(16)	28(7)	0	-5(7)	0
C3	48(10)	20(6)	35(10)	8(7)	4(8)	-5(7)
C4	18(3)	25(4)	44(4)	-2(3)	6(3)	-6(3)
C5	34(4)	29(4)	27(4)	-5(3)	2(3)	2(3)
C6	39(8)	17(8)	14(7)	-6(5)	-6(6)	-1(5)
C7	27(4)	35(4)	30(4)	-4(3)	-4(3)	-3(3)
C8	31(4)	28(4)	54(5)	9(4)	18(4)	12(3)
C9	500(60)	480(90)	240(50)	0	0	150(70)
C10	1050(80)	83(11)	200(30)	0	0	-140(20)

Table S24. Bond lengths for **bMOF-201.**

Atom	Atom	Length/Å	Atom	Atom	Length/Å
Zn1	O1	1.935(5)	N2	C6	1.584(15)
Zn1	N2	2.027(7)	N3	C1	1.358(13)
Zn1	N4[1]	1.964(6)	N3	C2	1.312(11)
Zn1	N5[2]	1.998(6)	N4	N5	1.400(8)
Zn2	O3	2.023(15)	N4	C7	1.327(9)
Zn2	O5	2.046(17)	N5	C5	1.321(9)
Zn2	N3	2.033(10)	C2	C3[4]	1.50(2)
Zn2	N3[3]	2.033(10)	C2	C3	1.50(2)
O1	C8	1.251(10)	C3	C3[4]	1.18(3)
O2	C8	1.246(10)	C3	C6	0.973(19)
O3	C10	1.23(2)	C3	C6[4]	1.387(19)
O4	C10	1.285(19)	C4	C5	1.389(10)
O5	C9	1.519(19)	C4	C7	1.381(10)
N1	C6	1.394(17)	C4	C8	1.508(10)
N2	C1	1.307(16)	C6	C6[4]	0.83(2)
N2	C3	1.288(17)			

[1]+X, +Z, 1/2-Y; [2] 1+Z, -1+X, +Y; [3] 2-X, -Y, +Z; [4] +X, -Y, +Z; [5] 2-X, +Y, +Z; [6] +X, 1/2-Z, +Y; [7] 1+Y, +Z, -1+X

Table S25. Bond Angles for **bMOF-201.**

Atom	Atom	Atom	Angle(°)	Atom	Atom	Atom	Angle(°)
O1	Zn1	N2	107.3(3)	$C3^{6}$	C2	C3	46.3(14)
O1	Zn1	$N4^{1}$	123.1(2)	N2	C3	C2	125.5(15)
O1	Zn1	$N5^{2}$	99.6(2)	N2	C3	$C6^{6}$	123.9(14)
$N4^{1}$	Zn1	N2	110.0(3)	$C3^{6}$	C3	N2	167.5(10)
$N4^{1}$	Zn1	$N5^{2}$	108.4(2)	$C3^{6}$	C3	C2	66.9(7)
$N5^{2}$	Zn1	N2	107.2(3)	$C3^{6}$	C3	$C6^{6}$	43.6(8)
O3	Zn2	O5	124.9(12)	C6	C3	N2	87.8(13)
O3	Zn2	N3	99.0(7)	$C6^{6}$	C3	C2	110.1(11)
O3	Zn2	$N3^{3}$	99.0(7)	C6	C3	C2	145.7(17)
N3	Zn2	O5	110.9(3)	C6	C3	$C3^{6}$	79.8(11)
$N3^{3}$	Zn2	O5	110.9(3)	C6	C3	$C6^{6}$	36.1(12)
$N3^{3}$	Zn2	N3	111.0(6)	C5	C4	C8	126.6(7)
C8	O1	Zn1	117.3(5)	C7	C4	C5	105.8(6)
C10	O3	Zn2	117(2)	C7	C4	C8	127.6(7)
C9	O5	Zn2	156(2)	N5	C5	C4	109.3(6)
C1	N2	Zn1	117.6(8)	$N1^{6}$	C6	N1	0.0(8)
C1	N2	C6	127.9(9)	$N1^{6}$	C6	N2	132.7(10)
C3	N2	Zn1	152.1(11)	N1	C6	N2	132.7(10)
C3	N2	C1	90.3(11)	$C3^{6}$	C6	$N1^{6}$	116.3(10)
C3	N2	C6	37.9(9)	$C3^{6}$	C6	N1	116.3(10)
C6	N2	Zn1	114.4(7)	C3	C6	N1	172.9(15)
C1	N3	Zn2	119.3(9)	C3	C6	$N1^{6}$	172.9(15)
C2	N3	Zn2	129.6(8)	$C3^{6}$	C6	N2	111.0(10)
C2	N3	C1	108.5(11)	C3	C6	N2	54.4(11)
N5	N4	$Zn1^{4}$	119.7(4)	C3	C6	$C3^{6}$	56.6(15)
C7	N4	$Zn1^{4}$	132.4(5)	$C6^{6}$	C6	$N1^{6}$	72.7(5)
C7	N4	N5	107.7(6)	$C6^{6}$	C6	N1	72.7(5)
N4	N5	$Zn1^{5}$	125.5(4)	$C6^{6}$	C6	N2	154.6(6)
C5	N5	$Zn1^{5}$	126.5(5)	$C6^{6}$	C6	$C3^{6}$	43.7(8)
C5	N5	N4	107.8(5)	$C6^{6}$	C6	C3	100.2(11)
N2	C1	N3	122.3(13)	N4	C7	C4	109.4(6)
N3	C2	$N3^{6}$	127.2(13)	O1	C8	C4	115.7(7)
N3	C2	$C3^{6}$	139.5(11)	O2	C8	O1	124.5(7)
$N3^{6}$	C2	C3	139.5(11)	O2	C8	C4	119.7(7)
N3	C2	C3	93.2(8)	O3	C10	O4	120(3)
$N3^{6}$	C2	$C3^{6}$	93.2(8)				

^{1}+X, +Z, 1/2-Y; ^{2} 1+Z, -1+X, +Y; ^{3} 2-X, -Y, +Z; ^{4} +X, -Y, +Z; ^{5} 2-X, +Y, +Z; ^{6} +X, 1/2-Z, +Y; ^{7} 1+Y, +Z, -1+X

Table S26. Torsion angles for **bMOF-201**.

A	B	C	D	Angle(°)	A	B	C	D	Angle(°)
Zn1	O1	C8	O2	-9.7(10)	C1	N2	C6	$N1^{1}$	-172.6(13)
Zn1	O1	C8	C4	169.2(5)	C1	N2	C6	$C3^{1}$	7.5(18)
Zn1	N2	C1	N3	-176.9(9)	C1	N2	C6	C3	7.2(18)
Zn1	N2	C3	C2	176.8(12)	C1	N2	C6	$C6^{1}$	8(2)
Zn1	N2	C3	$C3^{1}$	5(5)	C1	N3	C2	$N3^{1}$	-178.9(14)
Zn1	N2	C3	C6	6(3)	C1	N3	C2	C3	0.0(14)
Zn1	N2	C3	$C6^{1}$	6(3)	C1	N3	C2	$C3^{1}$	-1(3)
Zn1	N2	C6	N1	3.1(17)	C2	N3	C1	N2	-2.3(17)
Zn1	N2	C6	$N1^{1}$	3.1(17)	C2	C3	C6	N2	-167(4)
Zn1	N2	C6	$C3^{1}$	-176.8(10)	C2	C3	C6	$C3^{1}$	13(3)
Zn1	N2	C6	C3	-177.1(14)	C2	C3	C6	$C6^{1}$	13(3)
Zn1	N2	C6	$C6^{1}$	-176.4(9)	C3	N2	C1	N3	3.1(14)
$Zn1^{2}$	N4	N5	$Zn1^{3}$	-2.1(7)	C3	N2	C6	N1	-180(100)
$Zn1^{2}$	N4	N5	C5	173.7(5)	C3	N2	C6	$N1^{1}$	-180(100)
$Zn1^{2}$	N4	C7	C4	-174.0(5)	C3	N2	C6	$C3^{1}$	0.2(6)
$Zn1^{3}$	N5	C5	C4	178.2(5)	C3	N2	C6	$C6^{1}$	0.6(15)
Zn2	O3	C10	O4	180.0	$C3^{1}$	C2	C3	N2	-178.2(12)
Zn2	N3	C1	N2	161.4(8)	$C3^{1}$	C2	C3	$C6^{1}$	-5.9(15)
Zn2	N3	C2	$N3^{1}$	20(3)	$C3^{1}$	C2	C3	C6	-14(4)
Zn2	N3	C2	C3	-161.4(10)	$C3^{1}$	C3	C6	N2	179.7(7)
Zn2	N3	C2	$C3^{1}$	-162.0(17)	$C3^{1}$	C3	C6	$C6^{1}$	0.004(5)
N2	C3	C6	$C3^{1}$	-179.7(6)	C5	C4	C7	N4	-0.1(8)
N2	C3	C6	$C6^{1}$	-179.7(7)	C5	C4	C8	O1	6.0(11)
$N3^{1}$	C2	C3	N2	-179.0(18)	C5	C4	C8	O2	-175.0(7)
N3	C2	C3	N2	2(2)	C6	N2	C1	N3	-1.3(18)
N3	C2	C3	$C3^{1}$	-179.5(12)	C6	N2	C3	C2	171(3)
$N3^{1}$	C2	C3	$C3^{1}$	-0.8(19)	C6	N2	C3	$C3^{1}$	-1(3)
N3	C2	C3	C6	166(3)	C6	N2	C3	$C6^{1}$	-0.2(5)
$N3^{1}$	C2	C3	$C6^{1}$	-7(3)	$C6^{1}$	C3	C6	N2	179.7(7)
N3	C2	C3	$C6^{1}$	174.6(13)	$C6^{1}$	C3	C6	$C3^{1}$	-0.004(11)
$N3^{1}$	C2	C3	C6	-15(5)	C7	N4	N5	$Zn1^{3}$	-178.3(5)
N4	N5	C5	C4	2.4(8)	C7	N4	N5	C5	-2.5(8)
N5	N4	C7	C4	1.5(8)	C7	C4	C5	N5	-1.5(8)
C1	N2	C3	C2	-3.3(18)	C7	C4	C8	O1	-169.7(7)
C1	N2	C3	$C3^{1}$	-176(4)	C7	C4	C8	O2	9.3(12)
C1	N2	C3	$C6^{1}$	-174.5(16)	C8	C4	C5	N5	-178.0(7)
C1	N2	C3	C6	-174.3(14)	C8	C4	C7	N4	176.3(7)
C1	N2	C6	N1	-172.6(13)					

Table S27. Hydrogen atom coordinates (Å x 10^4) and isotropic displacement parameters (Å^2 x 10^3) for **bMOF-201.**

Atom	x	y	z	U(eq)
H5	3620(6)	5000	5000	535
H1A	2787.78	4817.69	3618.69	51
H1B	2623.55	5106.99	3594.66	51
H1	2958.57	5706.54	4459.73	98
H5A	3707.27	6020.91	3308.68	36
H7	3264.74	5528.55	2651.99	36
H9A	4110.79	4969.81	4957.78	610
H9B	4117.2	5248.37	5203.08	610
H9C	4118.88	5321.47	4839.13	610
H10	3606.35	5934.27	5000	534

Table S28. Atomic Occupancy for **bMOF-201**

Atom	Occupancy	Atom	Occupancy	Atom	Occupancy
H5	2	H1A	0.5	H1B	0.5
C3	0.5	C6	0.5	H9A	0.5
H9B	0.5	H9C	0.5		

Table S29. Solvent masks information for **bMOF-201**

Number	x	y	z	Volume	Electron count	Content
1	-0.753	-0.960	-0.173	34730.4	3276.6	?
2	0.000	0.000	0.250	11.6	2.0	?
3	0.000	0.000	0.750	11.6	2.0	?
4	0.000	0.250	0.000	11.6	2.0	?
5	0.000	0.250	0.500	11.6	2.0	?
6	0.000	0.500	0.250	11.6	2.0	?
7	0.000	0.500	0.750	11.6	2.0	?
8	0.000	0.750	0.000	11.6	2.0	?
9	0.000	0.750	0.500	11.6	2.0	?
10	0.250	0.000	0.000	11.6	2.0	?
11	0.250	0.000	0.500	11.6	2.0	?
12	0.250	0.500	0.000	11.6	2.0	?
13	0.250	0.500	0.500	11.6	2.0	?
14	0.500	0.000	0.250	11.6	2.0	?
15	0.500	0.000	0.750	11.6	2.0	?
16	0.500	0.250	0.500	11.6	2.0	?
17	0.500	0.250	0.000	11.6	2.0	?
18	0.500	0.500	0.250	11.6	2.0	?
19	0.500	0.500	0.750	11.6	2.0	?
20	0.500	0.750	0.000	11.6	2.0	?
21	0.500	0.750	0.500	11.6	2.0	?
22	0.750	0.000	0.000	11.6	2.0	?
23	0.750	0.000	0.500	11.6	2.0	?
24	0.750	0.500	0.500	11.6	2.0	?
25	0.750	0.500	0.000	11.6	2.0	?

References

1. S. Stoll and A. Schweiger, *J. Magn. Reson.*, 2006, **178**, 42-55.
2. J. VandeVondele, M. Krack, F. Mohamed, M. Parrinello, T. Chassaing and J. Hutter, *Comput. Phys. Commun., 2005***, 167** (2), 103-128.
3. J. P. Perdew, K. Burke and M. Ernzerhof, *Phys. Rev. Lett.,* 1996**, 77** (18), 3865-3868.
4. S. Grimme, J. Antony, S. Ehrlich and H. Krieg, *J. Chem. Phys.,* 2010**, 132** (15), 154104.
5. J. VandeVondele and J. Hutter, *J. Chem. Phys.*, 2007, **127**, 114105.
6. G. Henkelman and H. Jónsson, *J. Chem. Phys.,* 2000**, 113**, 9978.
7. G. Henkelman and H. Jónsson, *J. Chem. Phys.,* 2000**, 113**, 9901.
8. J. Wellendorff, K. T. Lundgaard, A. Møgelhøj, V. Petzold, D. D. Landis, J. K. Nørskov, T. Bligaard and K. W. Jacobsen, *Physical Review B*, 2012, **85**, 235149.
9. L. Gráfová, M. Pitoňák, J. Řezáč and P. Hobza, *Journal of Chemical Theory and Computation*, 2010, **6**, 2365-2376.
10. L. Li, Y. Yang, M. H. Mohamed, S. Zhang, G. Veser, N. L. Rosi and J. K. Johnson, *Organometallics*, 2019, **38**, 3453-3459.
11. G. C. Shearer, J. G. Vitillo, S. Bordiga, S. Svelle, U. Olsbye and K. P. Lillerud, *Chem. Mater.*, 2016, **28**, 7190-7193.
12. N. C. Jeong, B. Samanta, C. Y. Lee, O. K. Farha and J. T. Hupp, *J. Am. Chem. Soc.*, 2012, **134**, 51-54.
13. K. S. Park, Z. Ni, A. P. Côté, J. Y. Choi, R. Huang, F. J. Uribe-Romo, H. K. Chae, M. O'Keeffe and O. M. Yaghi, *Proc. Natl. Acad. Sci.*, 2006, **103**, 10186.
14. APEX2, SAINT, and SADABS; Bruker AXS Inc.: Madison WI, USA, 2014.
15. G.M. Sheldrick, *Acta Crystallogr.*, 2015**, A71**, 3-8.
16. G.M. Sheldrick, *Acta Crystallogr.*, 2015**, C71**, 3-8.
17. O. V. Dolomanov, L. J. Bourhis, R.J. Gildea, J. A. K. Howard and H. Puschmann, *J. Appl. Crystallogr.*, **2009,** 42, 339-341.
18. M. N. Burnett and C. K. Johnson, ORTEP-III: Oak Ridge Thermal Ellipsoid Plot Program for Crystal Structure Illustrations, Oak Ridge National Laboratory Report ORNL-6895, 1996.
